# A proof of concept ‘phase zero’ study of neurodevelopment using brain organoid models with Vis/near-infrared spectroscopy and electrophysiology

**DOI:** 10.1038/s41598-020-77929-8

**Published:** 2020-12-02

**Authors:** Anirban Dutta, Sneha Sudhakar Karanth, Mahasweta Bhattacharya, Michal Liput, Justyna Augustyniak, Mancheung Cheung, Ewa K. Stachowiak, Michal K. Stachowiak

**Affiliations:** 1 Department of Biomedical Engineering, University at Buffalo, Buffalo, 14260 USA; 2 Department of Pathology and Anatomical Sciences, University at Buffalo, Buffalo, 14260 USA; 3 Department of Stem Cells Bioengineering, Mossakowski Medical Research Centre, Polish Academy of Sciences, Warsaw, Poland; 4 Department of Neurochemistry, Mossakowski Medical Research Centre, Polish Academy of Sciences, Warsaw, Poland

**Keywords:** Diseases, Medical research, Neurology, Engineering

## Abstract

Homeostatic control of neuronal excitability by modulation of synaptic inhibition (I) and excitation (E) of the principal neurons is important during brain maturation. The fundamental features of in-utero brain development, including local synaptic E–I ratio and bioenergetics, can be modeled by cerebral organoids (CO) that have exhibited highly regular nested oscillatory network events. Therefore, we evaluated a 'Phase Zero' clinical study platform combining broadband Vis/near-infrared(NIR) spectroscopy and electrophysiology with studying E–I ratio based on the spectral exponent of local field potentials and bioenergetics based on the activity of mitochondrial Cytochrome-C Oxidase (CCO). We found a significant effect of the age of the healthy controls iPSC CO from 23 days to 3 months on the CCO activity (chi-square (2, N = 10) = 20, p = 4.5400e−05), and spectral exponent between 30–50 Hz (chi-square (2, N = 16) = 13.88, p = 0.001). Also, a significant effect of drugs, choline (CHO), idebenone (IDB), R-alpha-lipoic acid plus acetyl-l-carnitine (LCLA), was found on the CCO activity (chi-square (3, N = 10) = 25.44, p = 1.2492e−05), spectral exponent between 1 and 20 Hz (chi-square (3, N = 16) = 43.5, p = 1.9273e−09) and 30–50 Hz (chi-square (3, N = 16) = 23.47, p = 3.2148e−05) in 34 days old CO from schizophrenia (SCZ) patients iPSC. We present the feasibility of a multimodal approach, combining electrophysiology and broadband Vis–NIR spectroscopy, to monitor neurodevelopment in brain organoid models that can complement traditional drug design approaches to test clinically meaningful hypotheses.

## Introduction

Homeostatic control of neuronal excitability by modulation of synaptic inhibition (I) and excitation (E) of the principal neurons during brain maturation is important to avoid runaway excitability conditions^1^. The neuronal activity gives rise to transmembrane currents that can be measured in the extracellular medium^2^, where local synaptic E–I balance can be inferred non-invasively from local field potentials (LFPs)^3^. The power spectrum of LFPs has been reported to scale as the inverse of the frequency^4^, where Gao et al.^3^ showed that E–I changes could be estimated from the power-law exponent (or slope). Besides LFPs, the spiking output of neurons can also be detected in the extracellular medium^2^. In early brain development, high excitability of the principal neurons may be beneficial to respond to low-input currents^5^; then, the initial phase of postnatal brain development involves a rapid decrease in the E–I ratio with a disproportional increase of inhibitory conductance^1^. Prior work^6^ has also shown that this rapid neurodevelopment of cortical circuits occurs in an environment of altered energy availability. It can be postulated that the cortical circuit dynamics starts in a reverberating regime with a high E–I ratio, and then need to self-organize to different points in the reverberating regime^7^ with a decrease in the E–I ratio based on homeostatic plasticity, external inputs^8^, and the energy landscape of the developing brain^9^. Here, GABAergic inhibitory cells locally control the activity of numerous excitatory principal cells, so that the postnatal cortical circuit dynamics can maintain local E–I balance^10^. In fact, E–I balance has been postulated to be an organizing framework for investigating mechanisms in neuropsychiatric disorders^10^. Here, any runaway excitation or quiescence in brain development can lead to abnormal regulation of the E–I balance, which can lead to nervous system disorders, including schizophrenia^11^. In fact, in-utero brain development has been implicated in the pathophysiology of schizophrenia^12^; however, the first symptoms of schizophrenia have been shown to manifest only in early adulthood^13^. Here, recent works have shown that brain organoid models can provide an understanding of the fundamental features of in-utero neurodevelopment in health and disease^14^. In principal accordance, we present a 'Phase Zero' clinical study platform to investigate changes in the E–I ratio in conjunction with the bioenergetics in health and disease^15^.

Brain bioenergetics in health and disease is driven by mitochondrial function^15^. Mitochondria occupies about one-tenth of the total gray matter volume and plays an essential role in the synaptic information processing determining the neuronal performance^16,17^. Maintenance of the neuronal activity requires maintained energy supplies, including synaptic transmission – the main energy consumer^18^. The activity of mitochondrial Cytochrome-C Oxidase (CCO), an activator of adenosine triphosphate (ATP) synthase – a marker of mitochondrial function^19^, has been found higher in GABAergic neurons than in the surrounding pyramidal cells^20^. In fact, it has been shown that synaptic boutons with higher synaptic activity contained higher levels of respiratory chain protein cytochrome-c (CytC)^16^ that indicated that the synaptic performance needed to be locally matched with bioenergy supply. Also, mitochondria have been found to regulate neuronal energy metabolism^21,22^. CCO (also known as complex IV) is the terminal complex of the mitochondrial respiratory chain. CCO is comprised of 13 different subunits encoded by three mitochondrial genes (COX subunits I, II, and III) and ten nuclear genes (COX subunits IV, Va, Vb, VIa, VIb, VIc, VIIa, VIIb, VIIc, and VIII) for the molecular pathways involved in mitochondrial energy production. Energy cost in the cortical layers depends on the overall state of homeostatic regulation of global firing rates of neurons determined by the dynamic interaction between the principal cells and the interneurons^20^. Here, E–I ratio is postulated to be related to the CCO concentration that can be measured non-invasively using broadband (490–900 nm) spectroscopy in humans^19,23^, as well as in the intact cells and tissues in-vitro^24,25^ using a proposed 'Phase Zero' clinical study platform^26,27^. Bioenergetics is crucial during neurodevelopment since interneuron precursors need to migrate toward the neocortex and hippocampus^28^ that is postulated to be fueled by mitochondrial oxidative phosphorylation^29^. We leveraged partial least square processing of the Vis–NIR spectra using redox calibration data for the quantification of CCO activity in the cerebral organoids. In this proof-of-concept 'Phase Zero' study, we investigated CCO activity in the cerebral organoids during the initial phase of healthy neurodevelopment, where a decrease in the E–I ratio was expected^1^.

In schizophrenia, prior works^30–32^ have shown alterations of molecular pathways involved in mitochondrial energy production. Specifically, a defect in the mitochondrial oxidative phosphorylation^33^ with decreased activity of the electron transport chain (ETC) complexes, including the reduced activity of complex IV, has been observed^32^. Our cerebral organoid studies using induced pluripotent stem cells (iPSCs) from schizophrenia patients and healthy control individuals revealed improperly clustered immature neurons in cortical layers II, III, and V, and a decreased intracortical connectivity with disrupted orientation and morphology of calretinin interneurons^12^—a subpopulation of GABAergic cells. Since calretinin interneurons play a crucial role in the generation of synchronous, rhythmic neuronal activity by interacting with other interneurons^34^; therefore, we investigated the E–I ratio in conjunction with CCO activity in the cerebral organoids from schizophrenia patients and healthy controls. Here, GABAergic interneuron defects in schizophrenia^12^ were postulated to increase the power-law exponent and the E–I ratio (away from E–I balance hypothesis^10^) and reduce the signal-to-noise ratio (SNR) in the neuronal circuits^10^ that may be related to the bioenergetics^35^. Low SNR can make information processing less efficient^10^ that can manifest as cognitive deficits in early adulthood^13^. Importantly, antipsychotics are not very effective in reversing cognitive deficits in adulthood^36^. Moreover, dysfunctional calretinin interneurons can lead to increased excitatory transmission (increased E–I) and excitotoxicity, leading to delayed pyramidal cell death^37^. Here, characterizing the mechanisms of the pathogenesis of cognitive deficits in schizophrenia is crucial to develop drug treatments^36^. A recent study^36^ provided evidence that restoration of mitochondrial function was accompanied by a reversal of deficits in cortical interneurons in schizophrenia. It has been postulated that promoting mitochondrial function, including ETC, would rescue cortical interneurons towards healthy neurodevelopment in the brain susceptible to schizophrenia. Here, prior work^38^ on dietary phosphatidylcholine (CHO)^38^ supplementation starting in the second trimester has shown to activate the timely development of cerebral inhibition based on electrophysiological recordings. At the same time, Idebenone (IDB)^39^, and R-alpha-lipoic acid plus acetyl-L-carnitine (LCLA)^36,40^ are mitochondrial drugs that are proposed to have an effect on the neonatal pathophysiology related to later schizophrenia risk. In this proof-of-concept study, we investigated the feasibility of capturing drug effects of CHO^38^, IDB^39^, and LCLA^36,40^, on cerebral organoids from schizophrenia patients using a 'Phase Zero' clinical study platform.

## Methods

### Protocol for organoid development and drug treatment

Cerebral organoids were generated using two healthy control and two schizophrenic cell lines following previously established protocol^12^. The two commercial healthy control cell lines (23,476*C: Female and 3651: Female), and the two commercial schizophrenic cell lines (1835: Female and 1792: Male) were developed and characterized for human cerebral organoids in our prior studies^12,41,42^. These representative lines were selected from six control and four schizophrenic cell lines which showed consistent differences in neuronal development, and multi-gene transcriptomes^12,41,43^, and in organoid corticogenesis between the apparently healthy and diverse schizophrenic patients. To generate Embryoid Bodies (EBs), confluent induced Pluripotent Stem Cells (iPSC) were maintained on Vitronectin-coated surfaces in combination with Essential 8 (E8) medium in a feeder-free condition. They were then dissociated using EDTA and plated on a 6-well ultra-low attachment plate (Corning, USA) in E8 medium and maintained in the E8 media for 2–3 days. Then, the media was changed to N2/B27 media with dual SMAD inhibitors (which rapidly increases neural differentiation) exchanged daily for 3–4 days. Then, the EBs were transferred into a 24-well low attachment plate (1–2 EBs per well) and grown in neural induction medium for four days. To generate cerebral organoids, neurosperes/neuroepithelium (with visible bright marginal zones) were embedded in matrigel droplets and transferred to 6 cm plates in cerebral organoid media without vitamin A for four days, followed by transfer to an orbital shaker and in cerebral organoid media with vitamin A. After ten days in the shaker (14 days after matrigel embedding), the cerebral organoids were treated with (a) Vehicle (control), (b) Idebenone (IDB), (c) Lipoic acid + Acetyl l-carnitine (LCLA), and (d) choline (CHO). Media was changed every 2 days, and then the cerebral organoids at various timepoints of maturity were embedded to the center of 3.5 cm plate using matrigel, and the broadband Vis/near-infrared spectroscopy and electrophysiological investigation were performed. The time points were 34 days for the schizophrenic cerebral organoids, and 23 days, two months, three months for the healthy controls cerebral organoids.

### Experimental setup for broadband Vis/near-infrared spectroscopy and electrophysiological investigation

**Figure 1:**
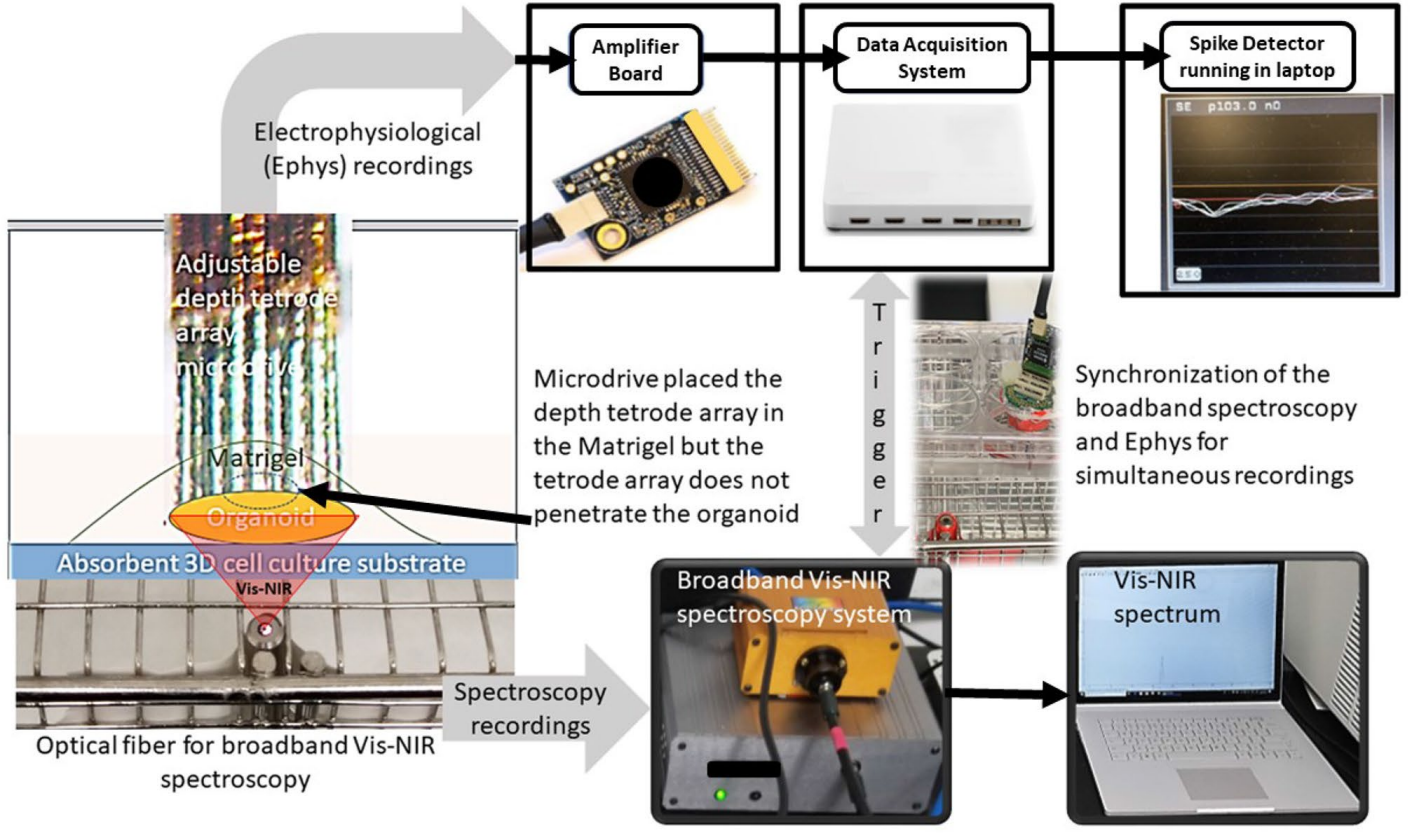
Experimental setup combining broadband vis/near-infrared spectroscopy and electrophysiology. The setup consists of a 32-channel tetrode microdrive integrated with the Intan RHD2132 amplifier (Intan Technologies, USA) and Open Ephys data acquisition system. Intan RHD2132 amplifier (Intan Technologies, USA) supports sampling 32 amplifier channels at 30 kSamples/s each, and provide fully integrated electrophysiology amplifier array with on-chip 16-bit analog-to-digital converter (ADC). We used the Open Ephys GUI and Spike Detector plugin for online monitoring of the data fidelity across the channels as well as for data recording in a laptop computer. Broadband Vis–NIR spectroscopy system consisted of the SILVER-Nova spectrometer (Stellarnet, USA) over the 190–1110 nm wavelength range with 200 μm slit for high sensitivity, SL1 high stability Tungsten Halogen light (350–2500 nm) source (Stellarnet, USA), and R600-8-VisNIR Reflectance Probe (Stellarnet, USA) for VIS–NIR spectroscopy with seven optical fibers bundled around one 600 μm fiber. We used the SpectraWiz spectrometer software (Stellarnet, USA) for recording the Broadband Vis–NIR spectroscopy data on a laptop computer.

Figure 1 shows the experimental setup that we developed for broadband Vis/near-infrared spectroscopy and electrophysiological investigation. The setup consisted of a 32-channel tetrode (eight polyimide-coated nickel-chrome tetrode wire of diameter ~ 50 µm arranged in a circular grid of ~ 2 mm diameter) microdrive^44^ integrated with Intan RHD2132 amplifier (Intan Technologies, USA) and Open Ephys data acquisition system^45^. The tetrode array was driven into the matrigel matrix^46^ in close proximity to the organoid surface using the microdrive (without penetrating the organoid). The electrical resistivity of the matrigel matrix was found^47^ to be comparable to the skin around 300 Ω.cm in the 100–1000 Hz range^48^. Intan RHD2132 amplifier (Intan Technologies, USA) supported sampling 32 amplifier channels at 30 kSamples/s each and provided a fully integrated electrophysiology amplifier array with on-chip 16-bit analog-to-digital converter (ADC). Open Ephys acquisition board can read from to 8 Intan amplifier chips using low-voltage differential signaling (LVDS) connected to a computer's USB port^45^. We used the Open Ephys GUI along with the LFP viewer and Spike Detector plugins to record in the fault-tolerant Open Ephys data format in blocks of 1024 samples, each of which included a timestamp and a readily identifiable 'record marker'^45^. Here, the network events related to the start trigger to synchronize with the broadband Vis–NIR spectroscopy system that was read from the TCP port. Broadband Vis–NIR spectroscopy system consisted of the SILVER-Nova spectrometer (Stellarnet, USA) over the 190–1110 nm wavelength range with 200um slit for high sensitivity, SL1 high stability Tungsten Halogen light (350-2500 nm) source (Stellarnet, USA), and R600-8-VisNIR Reflectance Probe (Stellarnet, USA) for VIS–NIR spectroscopy with seven optical fibers bundled around one 600 μm fiber. We used the SpectraWiz spectrometer software (Stellarnet, USA) for recording the Broadband Vis–NIR spectroscopy data in conjunction with the electrophysiological recording with Open Ephys GUI.

First, we calibrated the Broadband Vis–NIR spectroscopy system using a colorimetric assay kit (CYTOCOX1, Sigma-Aldrich, USA). We used the broadband (490–900 nm) spectroscopy for the determination of CCO activity (molecular weight 200,000 at pH 7^49^) in the standard 2 ml sample at various concentrations of 2.5 μg/ml, 1.25 μg/ml, 0.625 μg/ml, 0.3125 μg/ml, 0.1562 μg/ml, 0.0781 μg/ml, 0.05 μg/ml, 0.1 μg/ml, 0.15 μg/ml, 0.2 μg/ml, 0.25 μg/ml, 0.125 μg/ml, 0.175 μg/ml, as well as in the buffer solution. Principal component regression and partial least square processing of the Vis–NIR spectra were investigated to develop a multivariate regression model for the determination of the CCO activity in the organoid—see Appendix 1. We recorded spontaneous neuronal activity for a total of 10 min from 32-channel tetrode along with Vis–NIR spectroscopy in a staggered manner over ten trials each. The spontaneous neuronal activity is a combination of multi-unit activity (MUA) and LFPs that were saved by the Open Ephys GUI (Spike Detector plugin). The electrodes that did not record at least five spikes/min were discarded^50^. More than 50% of the electrodes recorded at least five spikes/min, so 16 best electrodes with most spikes/min were used for LFP analysis. LFPs were resampled at 500 Hz, and then processed by power spectral density (PSD) estimates computed using Welch's method with a window length of 2 s and overlap of 1 s^50^. Then, the spectral exponent^51^ of non-oscillatory PSD background was computed (fitPowerLaw3steps.m) from the PSD under the assumption that LFPs decays according to an inverse power-law: PSD(f) ∼1/f^α^. Here, the spectral exponent β = − α indexes the steepness of the decay of the PSD background, typically ranging from − 4 to − 1.5^52^. It is important to take the non-oscillatory PSD background for the power-law fitting (fitPowerLaw3steps.m) since a forward LFP computational model showed that the oscillatory peaks in the PSD could reduce the correlation of the power-law exponent with the E–I ratio^3^.

In our LFP recordings, both prominent oscillatory peaks, as well as aperiodic signal characteristic, have been observed^50^—see Appendix 2. Therefore, a three-step fitting procedure^51^ discarded the oscillatory peaks prior to estimating the background slope where fitting was done in 5 Hz windows. The average slope in the 30–50 Hz frequency range is postulated to be positively correlated with the synaptic E–I ratio based on forwarding LFP computational model^3^. Here, change in the slope at the higher frequency band (> 30 Hz) can occur separately than the change in the slope at a lower frequency band (< 30 Hz) based on prior work on the human cortex^53^. So, we also investigated the average slope in the lower frequency band of 1–20 Hz as a control since the lowest frequency oscillatory activity was primarily in the 10–15 Hz band^3^. We recorded from a 23 day old, two months old, and three months old cerebral organoids from healthy controls, and a 34 day old cerebral organoid from schizophrenia patients where it was postulated that older organoids from healthy controls would have a higher ratio of inhibitory neurons^50^ (i.e., lower E–I ratio and lower slope). Also, we investigated drug (CHO, IDB, LCLA) treated 34 days old cerebral organoids from schizophrenia patients, which was postulated to have a higher ratio of inhibitory neurons^50^ (i.e., lower E–I ratio, lower slope) when compared with the vehicle-treated control.

### Statistical analysis

In this proof of concept study, the statistical analysis was performed to determine the feasibility of our combined Vis/near-infrared spectroscopy and electrophysiology approach to capture the age effects of the healthy controls iPSC CO and the drug effects on the 34 days old CO from SCZ patients’ iPSC. We used a nonparametric version of the analysis of variance, nonparametric Friedman's test, that make only mild assumptions about the data, and are appropriate when the distribution of the data is non-normal. Specifically, Friedman’s tests the null hypothesis that the age effects of the healthy controls iPSC CO from 23 days to 3 months are all the same on the CCO activity and spectral exponent. Also, Friedman’s tests the null hypothesis that the drug (vehicle-treatment, CHO, IDB, LCLA) effects on the 34 days old CO from SCZ patients’ iPSC are all the same on the CCO activity and spectral exponent.

## Results

### Characterization of human cerebral organoids

The organoids formed polarized structures with forebrain-like upper region containing multiple rosettes, as shown in an example of DAPI stained control iPSC cerebral organoid in Appendix 4. Such polarized structures have been observed in previous studies^12,54^. At 5 weeks, we found that few rosettes of both control and schizophrenia organoids had residual lumen. The rosettes have been shown to contain proliferating Neuronal Stem/Progenitor cells^12,42^. The cortical zone has been shown to contain a network of neurons and underlying subcortical calretinin interneurons^12,42^.

### Electrophysiology and broadband Vis/near-infrared spectroscopy of the cerebral organoids without drug treatment

**Figure 2:**
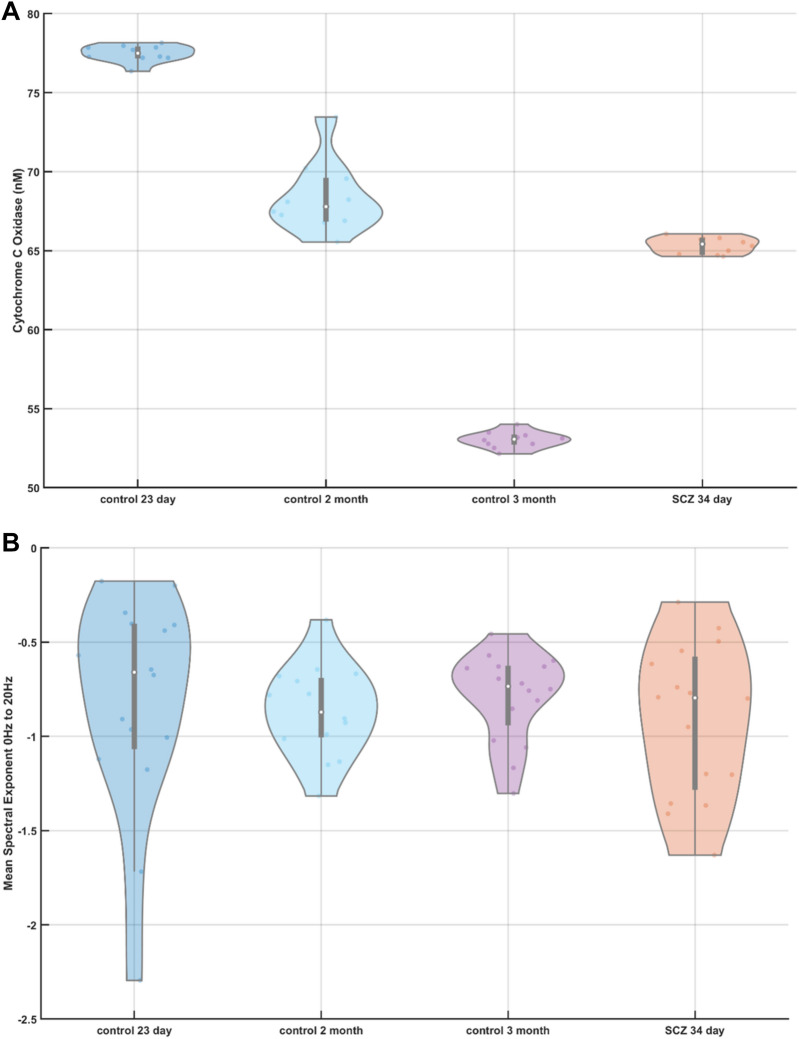

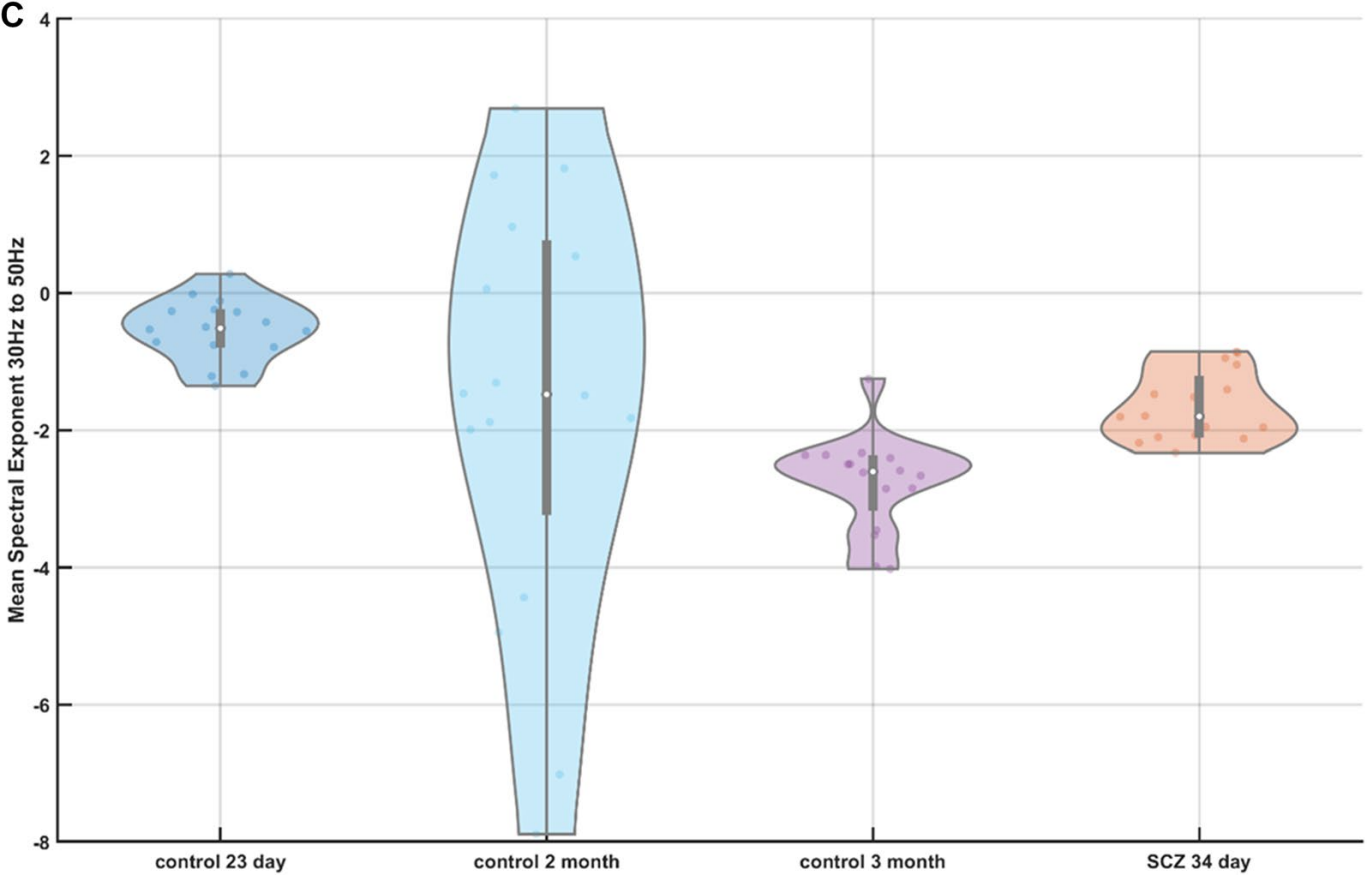
(**A**) Violin plot of the cytochrome C oxidase (CCO) activity across ten trials in cerebral organoids from healthy controls (control) at 23 days, two months, and three months, as well as a cerebral organoid from schizophrenia (SCZ) patients at 34 days (vehicle-treated). (**B**) Violin plot of the spectral exponent in the 1–20 Hz frequency band computed from 16 best (most spikes per min) tetrode recordings from cerebral organoids at 23 days, two months, and three months, for healthy controls (control) as well as a vehicle-treated cerebral organoid at 34 days from schizophrenia (SCZ) patients. (**C**) Violin plot of the spectral exponent in the 30–50 Hz frequency band computed from 16 best (most spikes per min) tetrode recordings from cerebral organoids at 23 days, two months, and three months, for healthy controls (control) as well as a vehicle-treated cerebral organoid at 34 days from schizophrenia (SCZ) patients. Violin plot allowed visualization of the distribution of the data and its probability density where the box plot (with median, interquartile range, upper adjacent value, lower adjacent value) is combined with the probability density placed on each side. A significant effect was found of the age of the healthy controls iPSC CO from 23 days to 3 months on the CCO activity (chi-square (2, N = 10) = 20, p = 4.5400e−05), and spectral exponent between 30–50 Hz (chi-square (2, N = 16) = 13.88, p = 0.001).

Multivariate regression modeling using partial least square regression (PLSR) as well as principal component regression (PCR) found that three components accounted for greater than 99% of the variance in the calibration data—see Fig. A1(A) in Appendix 1. Also, ten components accounted for 99.99% of the variance and provided an R-squared (the proportion of the variance in the dependent variable that is predictable from the independent variable) goodness-of-fit of 0.9906 for the PLSR model (compared to R-squared PCR10 = 0.9707—see Fig. A1(B) in Appendix 1). We used ten component PLSR model (PLS10—see Fig. A1(C) in Appendix 1) for estimating the CCO activity (in nM for molecular weight 200,000 at pH 7^49^) across ten trials, as shown in Fig. 2A as a violin plot. The violin plot allowed the visualization of the distribution of the data and its probability density where the box plot (with median, interquartile range, upper adjacent value, lower adjacent value) is combined with the probability density placed on each side. Friedman's test showed a significant (χ^2^ (2, N = 10) = 20, p = 4.5400e−05) effect of the age of the control cerebral organoids on the CCO activity. Here, the CCO activity decreased with increasing maturity of the cerebral organoids from healthy controls. The CCO activity in the vehicle-treated 34-day old cerebral organoids from the schizophrenia (SCZ) patients was found to be comparable to the CCO activity in the two months old healthy control. The corresponding average spectral exponents for the 16 best (most spikes per min) tetrode channels are shown as the violin plot in 1–20 Hz frequency band in Fig. 2B, and 30–50 Hz frequency band in Fig. 2C, which were calculated from the corresponding PSDs (shown in Appendix 2). Friedman's test showed an insignificant (χ^2^ (2, N = 16) = 3.13, p = 0.2096) effect of the age of the healthy cerebral organoids on the spectral exponent in 1–20 Hz frequency band; however, a significant (χ^2^ (2, N = 16) = 13.88, p = 0.001) effect of the age of the healthy cerebral organoids was on the spectral exponent in 30–50 Hz frequency band. Here, the spectral exponent in the 30–50 Hz frequency band decreased with increasing maturity of the cerebral organoids from the healthy controls, which indicated a decreased E–I ratio. Also, the spectral exponents of the vehicle-treated 34-day old cerebral organoids from the SCZ patients were found to be comparable to that of the two months old healthy control.

### Electrophysiology and broadband Vis/near-infrared spectroscopy of schizophrenia (SCZ) patients' cerebral organoids with drug treatment

**Figure 3:**
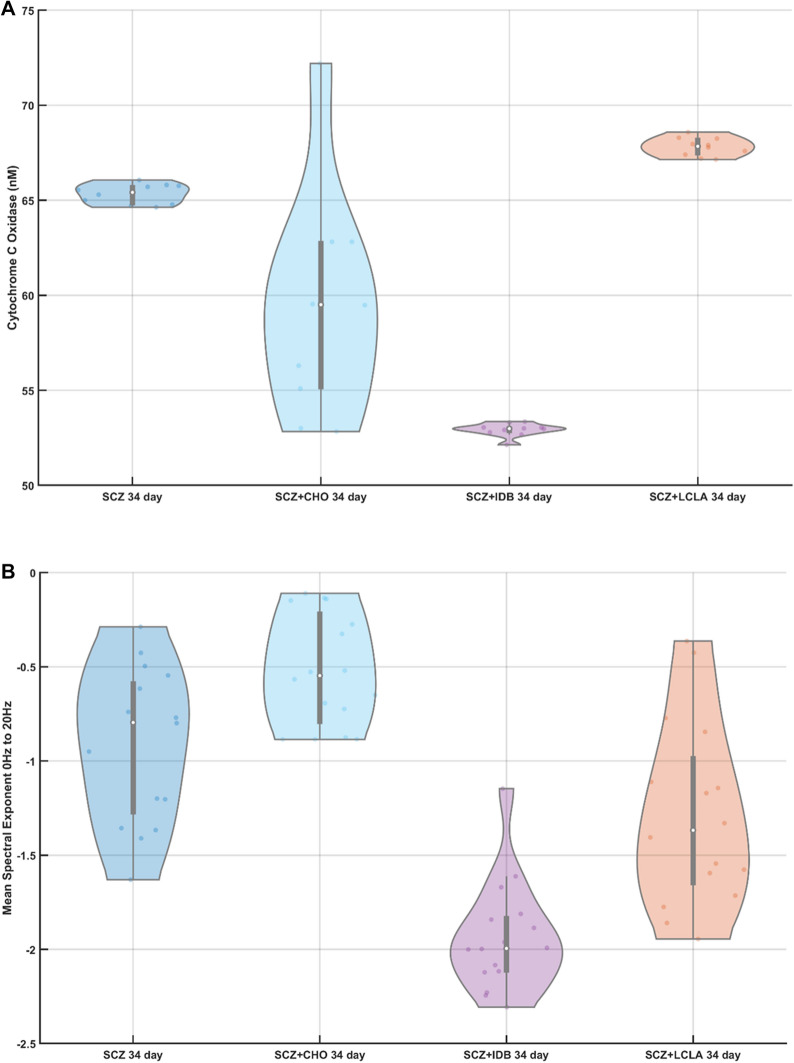

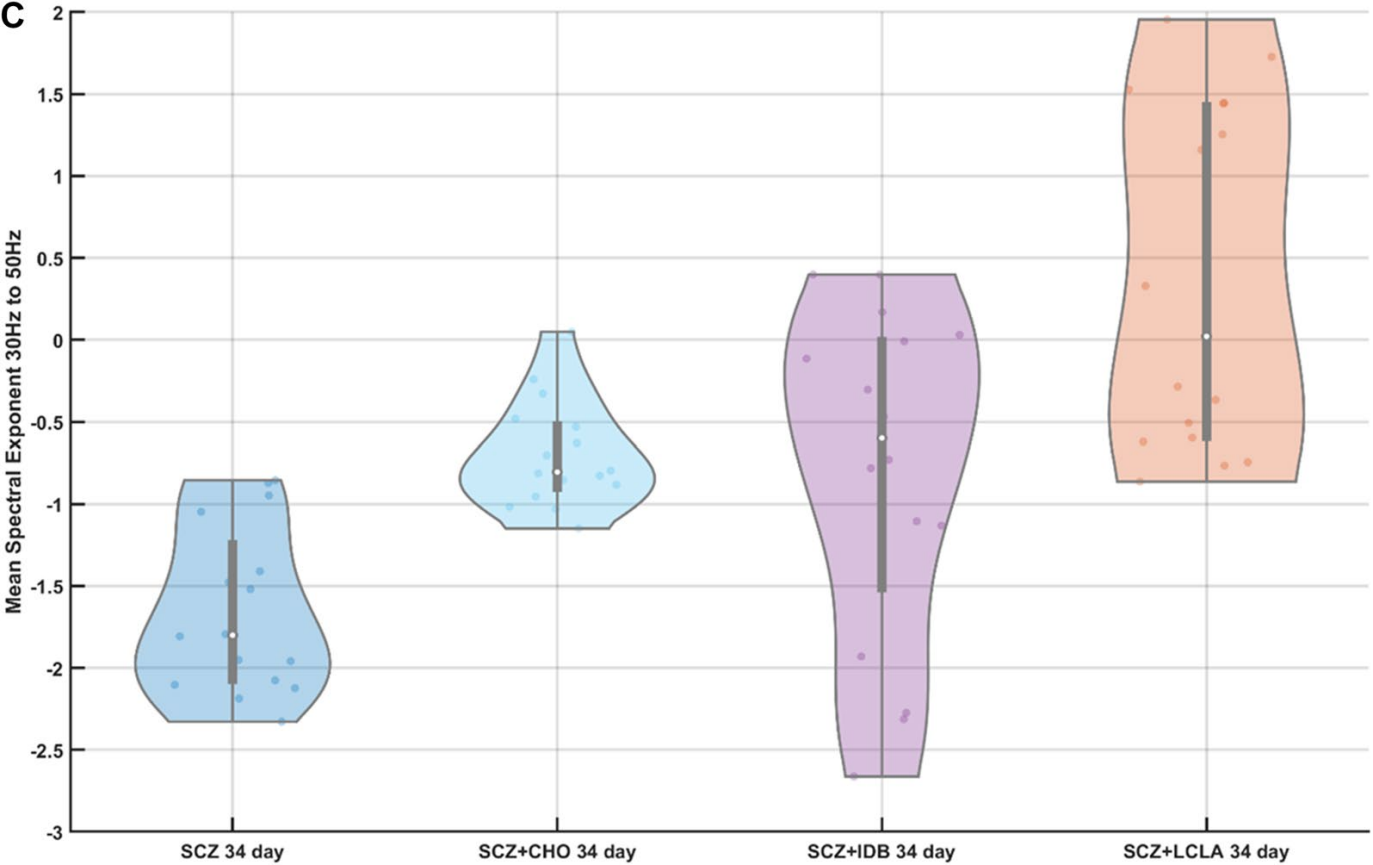
(**A**) Violin plot of the cytochrome C oxidase (CCO) activity across ten trials in 34 days old cerebral organoids from schizophrenia (SCZ) patients treated with vehicle and drugs, choline (CHO), idebenone (IDB), R-alpha-lipoic acid plus acetyl-L-carnitine (LCLA). (**B**) Violin plot of the spectral exponent in the 1–20 Hz frequency band computed for 16 best (most spikes per min) tetrode recordings from 34 days old cerebral organoids from schizophrenia (SCZ) patients, treated with vehicle and drugs, CHO, IDB, LCLA. (**C**) Violin plot of the spectral exponent in the 30–50 Hz frequency band computed for 16 best (most spikes per min) tetrode recordings from 34 days old cerebral organoids from schizophrenia (SCZ) patients, treated with vehicle and drugs, CHO, IDB, LCLA. Violin plot allowed visualization of the distribution of the data and its probability density where the box plot (with median, interquartile range, upper adjacent value, lower adjacent value) is combined with the probability density placed on each side. A significant effect was found of drugs, choline (CHO), idebenone (IDB), R-alpha-lipoic acid plus acetyl-L-carnitine (LCLA), was found on the CCO activity (chi-square (3, N = 10) = 25.44, p = 1.2492e−05), spectral exponent between 1–20 Hz (chi-square (3, N = 16) = 43.5, p = 1.9273e−09) and 30–50 Hz (chi-square (3, N = 16) = 23.47, p = 3.2148e−05) in 34 days old CO from schizophrenia (SCZ) patients iPSC.

We used a ten component PLSR model (PLS10) for estimating the CCO activity (in nM) across ten trials of 34-day old cerebral organoids from the schizophrenia (SCZ) patients with drug and vehicle treatment, as shown in Fig. 3A as violin plot. Friedman's test showed a significant (χ^2^ (3, N = 10) = 25.44, p = 1.2492e−05) effect of drugs on the CCO activity for the cerebral organoids from the SCZ patients. Here, the CCO activity decreased in the case of CHO and IDB while increased in the case of LCLA when compared to the vehicle-treated one. The corresponding average spectral exponents for the 16 best (most spikes per min) tetrode channels in 1–20 Hz frequency band and in the 30–50 Hz frequency band are shown as the violin plot in Fig. 2B and C respectively, which were calculated from the corresponding PSDs (shown in Appendix 2). The oscillatory peak between 10–15 Hz was found to be decreased by the drugs, especially in the case of IDB, when compared to the healthy controls—see Figure A2(D–G) (Appendix A2). Friedman's test showed a significant (χ^2^ (3, N = 16) = 43.5, p = 1.9273e−09) effect of drugs on the spectral exponent in the 1–20 Hz frequency band for the cerebral organoids from the SCZ patient. Also, Friedman's test showed a significant (χ^2^ (3, N = 16) = 23.47, p = 3.2148e−05) effect of drugs on the spectral exponent in 30 – 50 Hz frequency band; however, the effects were different from the 1–20 Hz frequency band. While the spectral exponent in the 1–20 Hz frequency band was decreased by IDB and LCLA, the spectral exponent increased in the 30–50 Hz frequency band when compared to the vehicle-treated one. CHO slightly increased the spectral exponent in both the frequency bands when compared to the vehicle-treated one.

We also investigated the effects of the drugs on the cerebral organoids from healthy controls that are presented in Appendix 3. All the three drugs reduced the CCO activity in the healthy controls; however, IDB and LCLA had opposite effects on the spectral exponent in the 30–50 Hz frequency band and no effect on the spectral exponent in the 1–20 Hz frequency band in the healthy controls when compared to the drug-treated SCZ cerebral organoids at 34 days. CHO had a similar effect on the spectral exponent in the 1–20 Hz frequency band; however, it increased the spectral exponent in the 30–50 Hz frequency band in healthy controls when compared to the vehicle-treated condition in the cerebral organoids at 2 months. In general, the spectral exponent in the 30–50 Hz frequency band was most responsive to the drug treatment.

## Discussion

In this proof-of-concept study, we showed the feasibility of a 'Phase Zero' clinical study platform to investigate changes in the E–I ratio based on the spectral exponent of LFPs in conjunction with changes in the bioenergetics based on the mitochondrial CCO activity. This proof-of-concept study assessed the feasibility of our combined Vis/near-infrared spectroscopy and electrophysiology approach, and not any clinically meaningful hypotheses^55^. This feasibility study provided preliminary evidence that showed a significant effect of the age of the healthy cerebral organoids on the spectral exponent in 30–50 Hz frequency band (χ^2^ (2, N = 16) = 13.88, p = 0.001) as well as on the CCO activity (χ^2^ (2, N = 10) = 20, p = 4.5400e−05) based on Friedman's test. Here, the spectral exponent in the 30–50 Hz frequency band was found (see Fig. 2C) to decrease with increasing maturity of the cerebral organoids in the healthy controls, which indicated a decrease in the E–I ratio in the early phase of healthy neurodevelopment^1^. This interpretation of the E–I ratio from the spectral exponent of LFPs is based on a published computational model^3^, where E–I ratio from 1:2 to 1:6 correlated positively with the PSD slope of the LFPs between 30 and 50 Hz. In the current in-vitro study, the spectral exponent in the 30–50 Hz frequency band reached a median around − 2.5 at three months (see Fig. 2C); however, Beggs and Plenz^56^ showed that the propagation of spontaneous activity obeys a power law with an exponent of − 1.5 for event sizes when close to the critical dynamics. During neurodevelopment, cerebral organoids exhibited periodic and highly regular nested oscillatory network events^50^; and, the difference between in-vitro and in-vivo power-law exponents is postulated due to a lack of homeostatic plasticity^57^ in-vitro that needs to be driven by external inputs^8^ to transition to a more in-vivo cortical circuit dynamics. Beggs and Plenz^56^ presented other works that have shown an exponent around − 1.5 to be characteristic of the cortical circuit dynamics. Here, an optimal value of E–I ratio (i.e., E–I balance) has been proposed to be determined following healthy neurodevelopment by the (near) criticality of the cortical circuit dynamics, which is a regime that maximizes information processing^7,56,58,59^. Additionally, we found that CCO activity decreased with increasing maturity of the cerebral organoids from healthy controls, as shown in Fig. 2B. This is postulated to be due to an increased inhibition in the cortical circuit dynamics, as found from a decrease in the spectral exponent in the 30–50 Hz frequency band (see Fig. 2C), with increasing maturity of the cerebral organoids. During healthy neurodevelopment, a metabolic shift of cells from glycolysis to oxidative phosphorylation is necessary for bioenergetics^60,61^, and for orchestrating nuclear transcriptional program^62,63^. When comparing the CCO activity as well as the spectral exponents of the vehicle-treated 34-day old cerebral organoids from the SCZ patients with that from healthy controls, we found 34-day old SCZ cerebral organoids comparable to two-months-old healthy cerebral organoids. In the absence of age-matched healthy controls, we can only postulate that the vehicle-treated 34-day old SCZ cerebral organoids suffered a faster decrease in the CCO activity and E–I ratio when compared to healthy controls, possibly due to excitotoxic cell death^37^—see Fig. 2B and C. Here, energy supply from mitochondria is essential and can be limiting for synaptic activity during neurodevelopment in schizophrenia, which may reciprocally modulate the motility and fusion/fission balance of mitochondria in the dendrites^22^ as well as homeostatic plasticity^64^. In fact, Ma et al.^58^ showed that the criticality in excitatory networks is established by the inhibitory plasticity and its circuit architecture that is fueled by mitochondrial oxidative phosphorylation^29^.

Our proof-of-concept study did not aim to determine clinically meaningful hypotheses^55^ of CHO^38^, IDB^39^, and LCLA^36,40^ drug effects on cerebral organoids from schizophrenia patients. Nevertheless, our provided insights into the feasibility of our combined Vis/near-infrared spectroscopy and electrophysiology approach to measure drug effects on cerebral organoids from the SCZ patients based on Friedman's test that showed a significant effect (χ^2^ (3, N = 10) = 25.44, p = 1.2492e−05) on the CCO activity that decreased in the case of CHO and IDB while increased in the case of LCLA when compared to the vehicle-treated one. This indicated the restoration of mitochondrial function by LCLA in agreement with prior work^36^. Furthermore, preliminary evidence based on Friedman's test showed a significant effect of drugs on the spectral exponent in the 1–20 Hz frequency band (χ^2^ (3, N = 16) = 43.5, p = 1.9273e−09) as well as 30–50 Hz frequency band (χ^2^ (3, N = 16) = 23.47, p = 3.2148e−05) of cerebral organoids from the SCZ patients; however, the effects were different for different drugs. While the spectral exponent in the 1–20 Hz frequency band was decreased by IDB and LCLA, the spectral exponent increased in the 30–50 Hz frequency band for the same drugs when compared to the vehicle-treated one. The increased spectral exponent in the 30–50 Hz frequency band and decreased spectral exponent in the 1–20 Hz frequency band may be due to the reversal of arborization deficits in cortical inhibitory neurons^36^ that needs further investigation in the future based on microscopy. In contrast, CHO slightly increased the spectral exponent in both the frequency bands when compared to the vehicle-treated one. Therefore, the spectral exponent increased in the 30–50 Hz frequency band for all the three drugs, which may be related to an increase in the E–I ratio^3^ in the drug-treated cerebral organoids from the SCZ patients when compared to the vehicle-treated one. However, CCO activity decreased in the case of CHO and IDB while increased in the case of LCLA when compared to vehicle-treatment. These results may reflect the underlying mechanism of action of different drugs on the E–I ratio and CCO activity that necessitates a larger sample size for testing clinically meaningful hypotheses^55^. The contrasting effect of CHO, when compared to IDB and LCLA, was found in the cerebral organoids from healthy controls at two months that was comparable in maturity to the 34-day old SCZ cerebral organoids (see Fig. 2). Here, we found that CHO significantly reduced the CCO activity in the healthy cerebral organoid at two months when compared to the vehicle-treatment condition (Appendix 3, Fig. A3.1(A)) in agreement with the SCZ results. However, CHO effects on the spectral exponent in the 1–20 Hz frequency band were different in the healthy controls than SCZ. Here, CHO did not significantly affect the spectral exponent in the 1 – 20 Hz frequency band (Appendix 3, Fig. A3.1(B)) but increased the spectral exponent in the 30–50 Hz frequency band (Appendix 3, Fig. A3.1(C)) when compared to the vehicle-treatment condition in the healthy cerebral organoid at two months. A similar effect was found in the CHO treated cerebral organoids from SCZ patients at 34 days when compared to the vehicle-treatment condition (see Fig. 3A). Therefore, CHO reduced the CCO activity while increasing the oscillatory activity in the 30–50 Hz frequency band in healthy cerebral organoids that was similar to SCZ results.

Limitations of this proof-of-concept study is small sample size such that this feasibility study was not statistically powered for testing clinically meaningful hypotheses^55^ on drug treatments in schizophrenia. So, our proof-of-concept study descriptively assessed the feasibility of our method^55^ where we used two healthy control lines and two schizophrenia lines from our prior published works^12,41,42^. We evaluated the CCO activity using broadband (490–900 nm) absorption spectroscopy, although the state of other complexes in the mitochondrial respiratory chain (complexes I–IV) may also be relevant in the drug effects. For example, IDB mediates electron transfer to complex III in the mitochondrial inner membrane, which was observed in this study based on the state of the complex IV since IDB can restore oxygen consumption at complex IV (and ATP production)^65^. Also, LCLA treatment has been shown to lead to the recovery of the activity of complex I and IV^40^. Since CCO is the terminal enzyme of the mitochondrial respiratory chain, so the activity of CCO can provide a measure of bioenergetics (ATP production). Here, ATP production by mitochondria at the pre-synapse and the post-synapse supplies energy for the synaptic transmission at the dendrites where the interactions are in both directions^22^. Although synaptic activity, as well as spikes together, can provide the energy budget^66^; however, we only investigated the synaptic E–I ratio in this study that was measured based on LFPs' spectral exponent. Here, the unidimensional E–I balance hypothesis may provide an incomplete picture, as found from the drug responses in this study, where more dimensions, including neural firing rates and correlations, may be relevant^67^. Nevertheless, most brain energy is used on synaptic transmission^18^, and neuropsychiatric disorders frequently involve deficits in synaptic E–I balance^10^ and synaptic energy supply^18^ so they were found crucial for this proof-of-concept study.

## Supplementary information

Supplementary Information.

## Data Availability

Data available on request from the authors.
