# Supplementary material for: A proof of concept ‘phase zero’ study of neurodevelopment using brain organoid models with Vis/near-infrared spectroscopy and electrophysiology
Source: Sci Rep. 2020 Dec 2;10:20987. doi: 10.1038/s41598-020-77929-8 (PMC7710726; doi:10.1038/s41598-020-77929-8)
Supplement: Supplementary file 1 — Supplementary Information. [file 41598_2020_77929_MOESM1_ESM.pdf]

# A proof of concept 'phase zero' study of neurodevelopment using brain organoid models with Vis/near-infrared spectroscopy and electrophysiology

Anirban Dutta[1,2*], Sneha Sudhakar Karanth[1,ⱡ], Mahasweta Bhattacharya[1,ⱡ], Michal Liput[2,3,ⱡ], Justyna Augustyniak[2,4,ⱡ], Mancheung Cheung[1], Ewa K. Stachowiak[2], Michal K. Stachowiak[1,2*]

[1] Department of Biomedical Engineering, University at Buffalo, Buffalo, 14260, USA.

[2] Department of Pathology and Anatomical Sciences, University at Buffalo, Buffalo, 14260, USA.

[3] Department of Stem Cells Bioengineering, Mossakowski Medical Research Centre, Polish Academy of Sciences, Warsaw, Poland

[4] Department of Neurochemistry, Mossakowski Medical Research Centre, Polish Academy of Sciences, Warsaw, Poland

[*]adutta@case.edu

*mks4@buffalo.edu

ⱡthese authors contributed equally to this work

In this proof-of-concept study, we found the feasibility of broadband Vis/near-infrared spectroscopy and electrophysiology during drug treatment of the cerebral organoids that provided preliminary insights into their different effects on age-matched neurodevelopment in healthy controls and SCZ patients (see Appendix 3). We found that IDB-treated healthy cerebral organoids at 34 days had a decreased CCO activity when compared to SCZ cerebral organoids (Appendix 3, Figure A3.2(A)). The effect of IDB on the spectral exponent in the 1 – 20Hz frequency band was found similar (Appendix 3, Figure A3.2(B)); however, the spectral exponent in the 30 – 50Hz frequency band increased in the case of healthy controls when compared to SCZ cerebral organoid (Appendix 3, Figure A3.2(C)). LCLA treatment decreased the CCO activity in the healthy cerebral organoid at 34 days when compared to SCZ cerebral organoids (Appendix 3, Figure A3.3(A)). Also, the effect of LCLA on the spectral exponent in the 1 – 20Hz frequency band was found similar (Appendix 3, Figure A3.3(B)); however, the spectral exponent in the 30 – 50Hz frequency band decreased in the case of healthy controls when compared to SCZ patients (Appendix 3, Figure A3.3(C)). Here, IDB and LCLA had opposite effects on the E-I ratio in healthy controls when compared to SCZ cerebral organoids at 34 months, where IDB increased while LCLA decreased the E-I ratio. Also, both IDB and LCLA led to a decrease in the CCO activity in healthy controls when compared to SCZ patients. Therefore, the effects on the age-matched healthy controls were found different for IDB and LCLA, which should be taken into account in future studies. Here, IDB drug treatment decreased the CCO activity and the spectral exponent in the 1 – 20Hz frequency band when compared to the vehicle-treatment in the cerebral organoids from SCZ patients. CHO also decreased the CCO activity; however, it increased the spectral exponent in both the frequency bands, which can be related to the reversal of impaired recurrent inhibition[1,2] in the cerebral organoids from SCZ patients. We also found that CHO effects on the spectral exponent in the 1 – 20Hz frequency band were different in the healthy controls than SCZ, which may be due to lack of related deficits in the arborization and recurrent inhibition in the healthy controls. Indeed, distinct inhibitory circuits orchestrate the oscillations in the cortical circuits[3], which complicates the E-I balance hypothesis for pharmacology where the relative activity of different subtypes of excitatory or inhibitory neurons can be affected differently by the drugs[4]. In the current study, we investigated the spectral exponent[5] of non-oscillatory PSD background; however, the oscillatory activity in the PSD may also be relevant. Here, all the drugs were found to reduce the oscillatory activity in the 10 – 15Hz frequency band when compared to vehicle-treatment conditions (Appendix 2, Figure A2(D)). Also, CHO was found to enhance the oscillatory activity between 30 – 35Hz (Appendix 2, Figure A2(E)) while reducing CCO activity. IDB that affects mitochondrial bioenergetics[39] (see Figure 3(A)) by mediating electron transfer to complex III in the mitochondrial inner membrane[60] was found to silence all oscillatory activity (Appendix 2, Figure A2(F)) while decreasing CCO activity. LCLA has been shown to act on the activities of the mitochondrial complexes[1,6] that increased the CCO activity, which may be related to the high-frequency broadband activity between 35 – 45Hz (Appendix 2, Figure A2(G)). Such changes in the high-frequency components in the LFPs may represent aggregate spiking activity of the local fast-spiking inhibitory neuronal population[7], which may be relevant in addition to the changes in the spectral exponent from non-oscillatory PSD background that was investigated in the current study. Future studies can investigate the mechanism of action using a multidimensional approach combining features from electrophysiology (e.g., synaptic E-I balance[4] and the distributions of spike covariances[8]) and time-resolved vibrational spectroscopy and other techniques[9] that can complement traditional drug design approaches, which are based on reversing molecular deficits, to push the system towards normative parameter space. Also, spectrophotometric assays can be used to measure the enzymatic activities of all the complexes I–IV[10],[11] for a more complete investigation. Furthermore, future studies need to investigate closed-loop non-invasive organoid stimulation[12] as external inputs[13] to longitudinally modulate E-I balance in the cerebral organoid's functional circuits[14], which may be necessary to transition to a more in-vivo cortical circuit dynamics[15].

S1. Ni, P. *et al.* iPSC-derived homogeneous populations of developing schizophrenia cortical interneurons have compromised mitochondrial function. *Molecular Psychiatry* 1–16 (2019) doi:10.1038/s41380-019-0423-3.

S2. Albiñana, E. *et al.* Choline induces opposite changes in pyramidal neuron excitability and synaptic transmission through a nicotinic receptor-independent process in hippocampal slices. *Pflugers Arch.* **469**, 779–795 (2017).

S3. Chen, G. *et al.* Distinct Inhibitory Circuits Orchestrate Cortical beta and gamma Band Oscillations. *Neuron* **96**, 1403-1418.e6 (2017).

S4. Sohal, V. S. & Rubenstein, J. L. R. Excitation-inhibition balance as a framework for investigating mechanisms in neuropsychiatric disorders. *Molecular Psychiatry* **24**, 1248–1257 (2019).

S5. Colombo, M. A. *et al.* The spectral exponent of the resting EEG indexes the presence of consciousness during unresponsiveness induced by propofol, xenon, and ketamine. *NeuroImage* **189**, 631–644 (2019).

S6. Long, J. *et al.* Mitochondrial decay in the brains of old rats: ameliorating effect of alpha-lipoic acid and acetyl-L-carnitine. *Neurochem. Res.* **34**, 755–763 (2009).

S7. Buzsáki, G., Anastassiou, C. A. & Koch, C. The origin of extracellular fields and currents--EEG, ECoG, LFP and spikes. *Nat. Rev. Neurosci.* **13**, 407–420 (2012).

S8. Wilting, J. & Priesemann, V. 25 years of criticality in neuroscience — established results, open controversies, novel concepts. *Current Opinion in Neurobiology* **58**, 105–111 (2019).

S9. Nakashima, S., Ogura, T. & Kitagawa, T. Infrared and Raman spectroscopic investigation of the reaction mechanism of cytochrome c oxidase. *Biochimica et Biophysica Acta (BBA) - Bioenergetics* **1847**, 86–97 (2015).

S10. Janssen, A. J. M. *et al.* Spectrophotometric assay for complex I of the respiratory chain in tissue samples and cultured fibroblasts. *Clin. Chem.* **53**, 729–734 (2007).

S11. Spinazzi, M., Casarin, A., Pertegato, V., Salviati, L. & Angelini, C. Assessment of mitochondrial respiratory chain enzymatic activities on tissues and cultured cells. *Nat Protoc* **7**, 1235–1246 (2012).

S12. Dagar, S., Chowdhury, S. R., Bapi, R. S., Dutta, A. & Roy, D. Near-Infrared Spectroscopy – Electroencephalography-Based Brain-State-Dependent Electrotherapy: A Computational Approach Based on Excitation–Inhibition Balance Hypothesis. *Front Neurol* **7**, (2016).

S13. Zierenberg, J., Wilting, J. & Priesemann, V. Homeostatic Plasticity and External Input Shape Neural Network Dynamics. *Phys. Rev. X* **8**, 031018 (2018).

S14. Bhattachar, M., Dutta, A., Freedman, D., Stachowiak, E. & Stachowiak, M. *Development of bidirectional 'mini-Brain' computer interface (mBCI) to modulate functional neural circuits – stimulation and recording from a cerebral organoid*. (2014). doi:10.13140/RG.2.2.21380.78728.

S15. Tien, N.-W. & Kerschensteiner, D. Homeostatic plasticity in neural development. *Neural Development* **13**, 9 (2018).

# Appendix 1

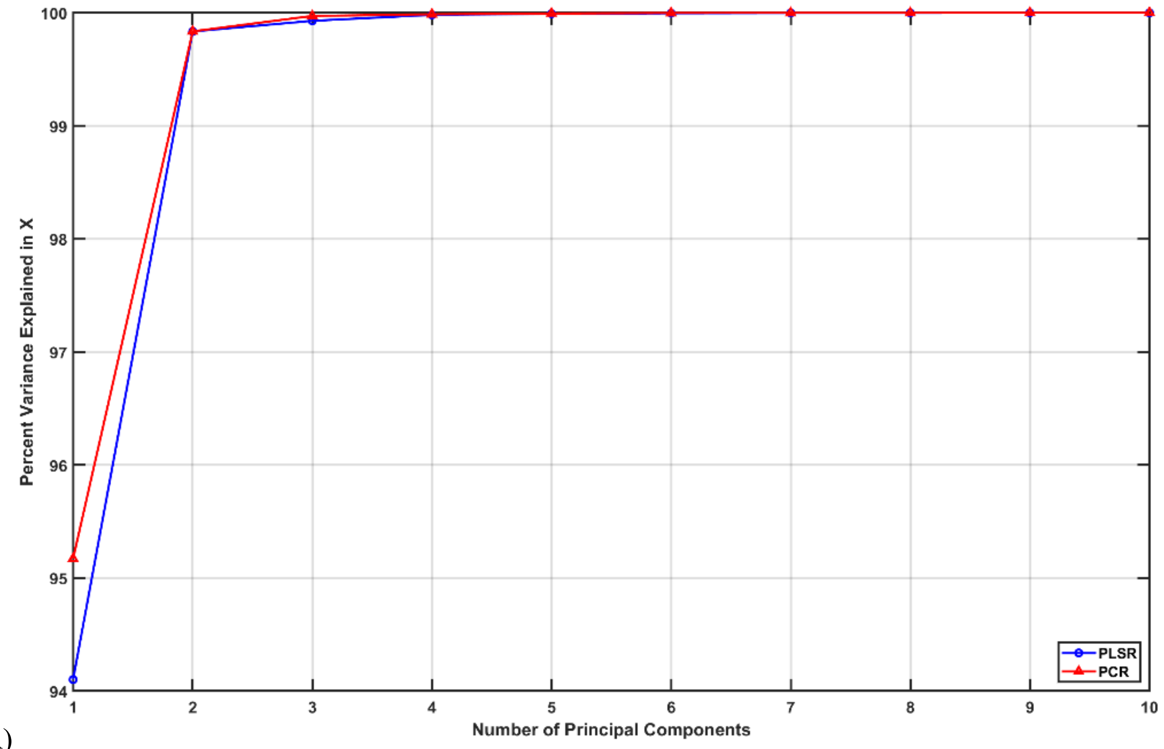


(A)

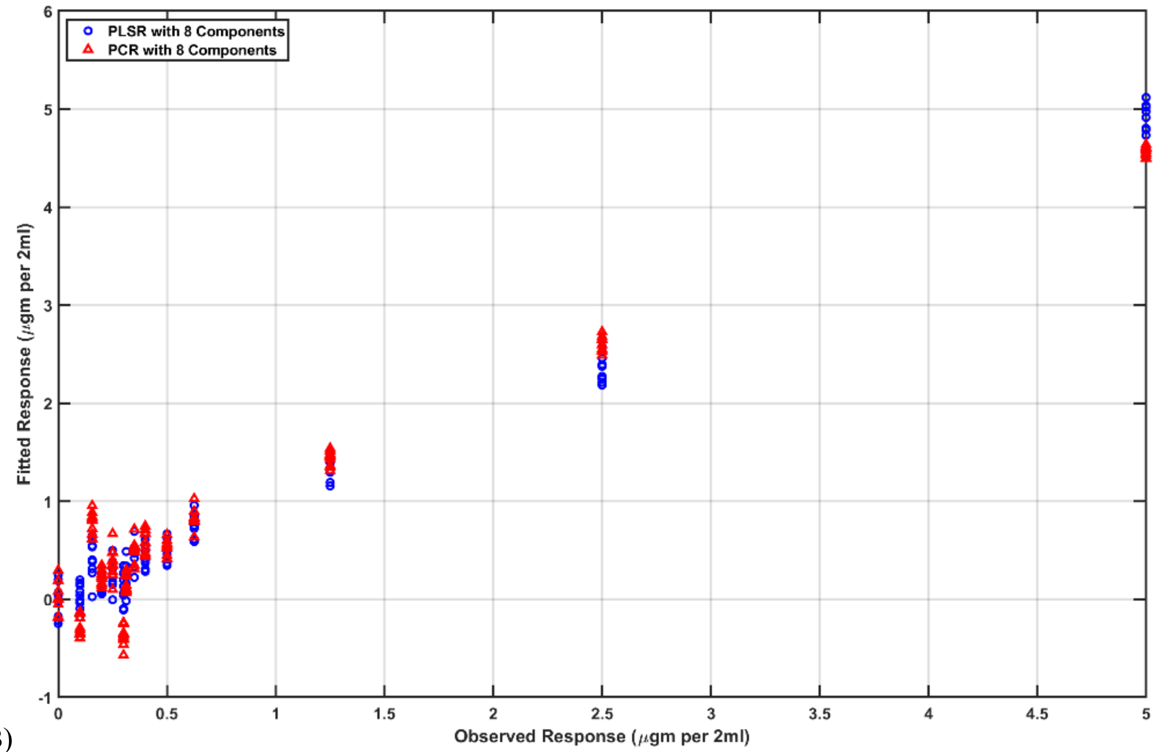


(B)

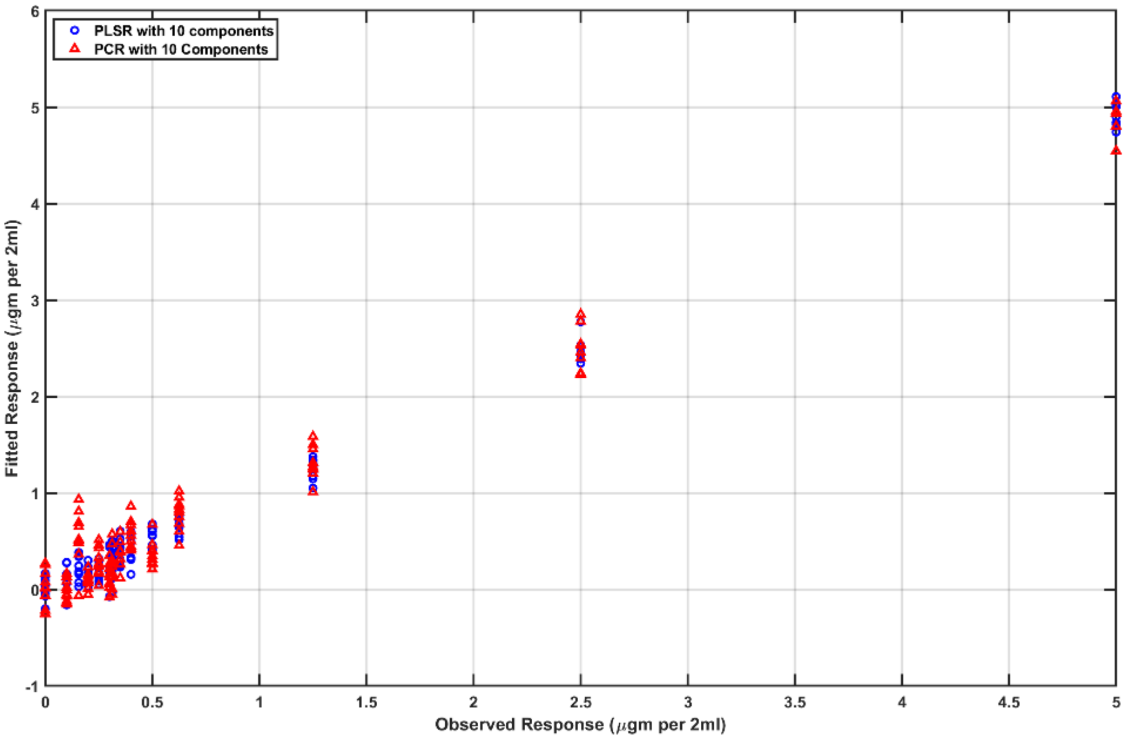


(C)

Figure A1: (A) Variance accounted for by the first ten components from principal component regression (PCR) and partial least square regression (PLSR) of the Vis-NIR spectra, (B) Observed response versus fitted response using first eight components only from PCR and PLSR. R-squared PCR8=0.9363, R-squared PCR10=0.9707, (C) Observed response versus fitted response using the first ten components only from PCR and PLSR. R-squared PLS8=0.9816, R-squared PLS10=0.9906.

## Appendix 2

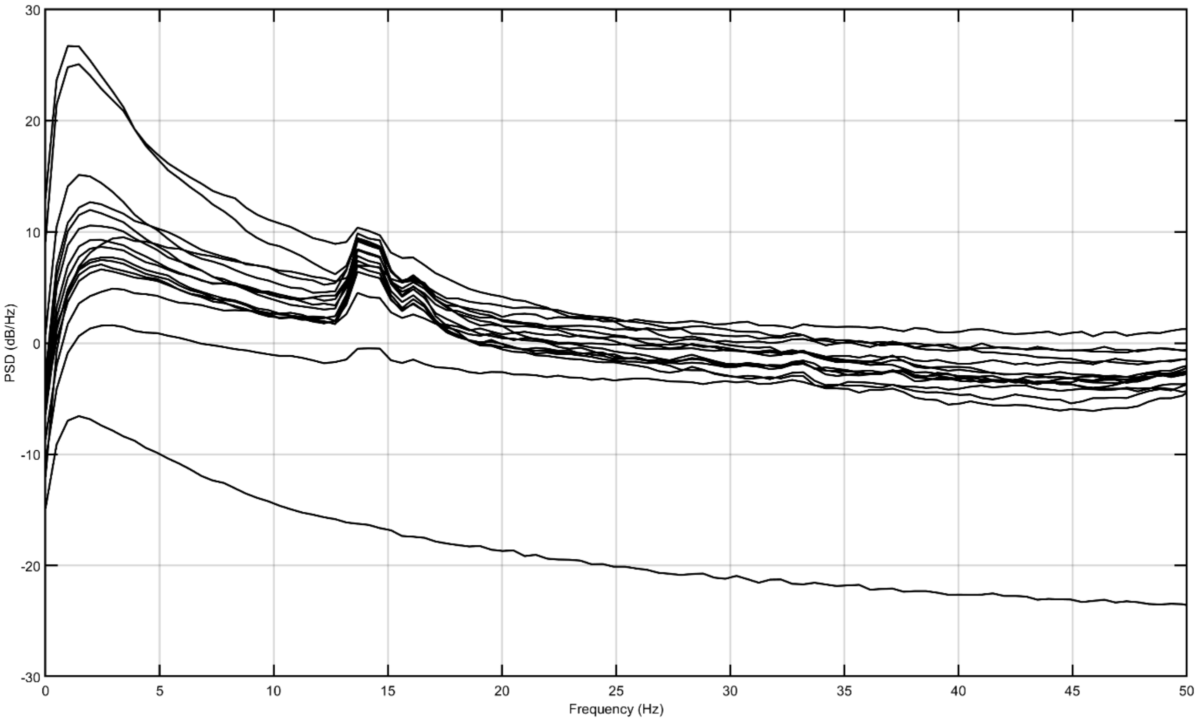


(A)

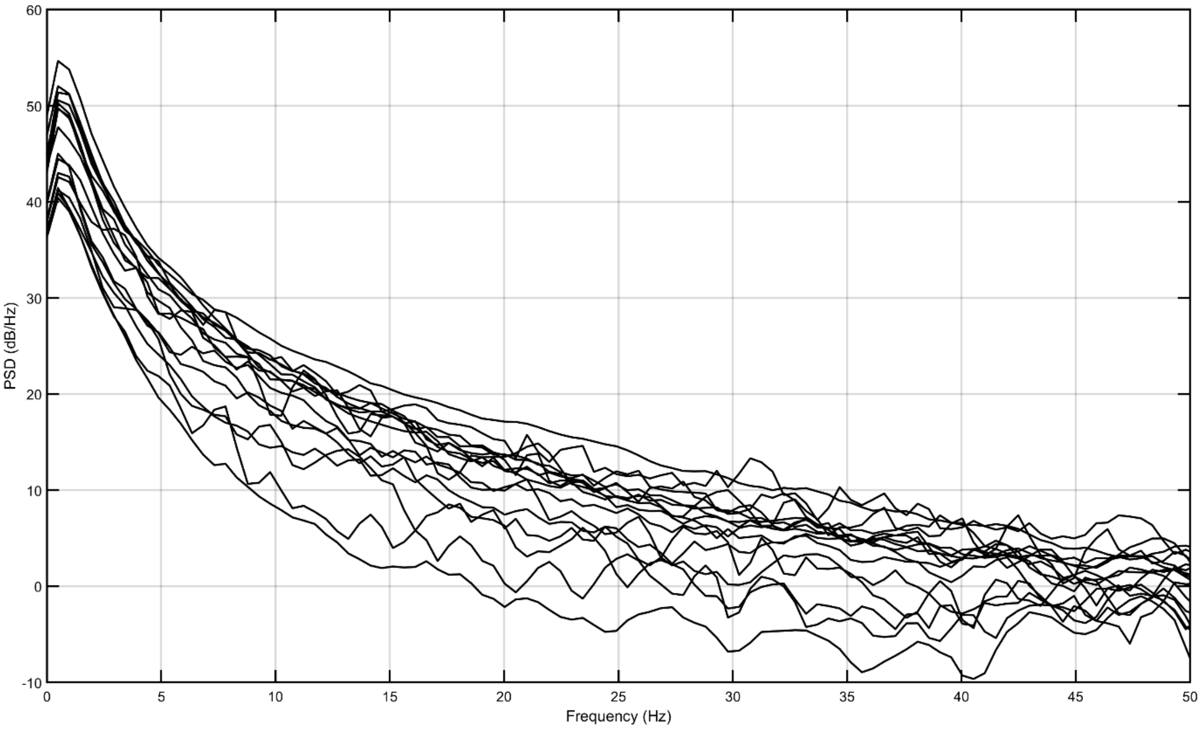


(B)

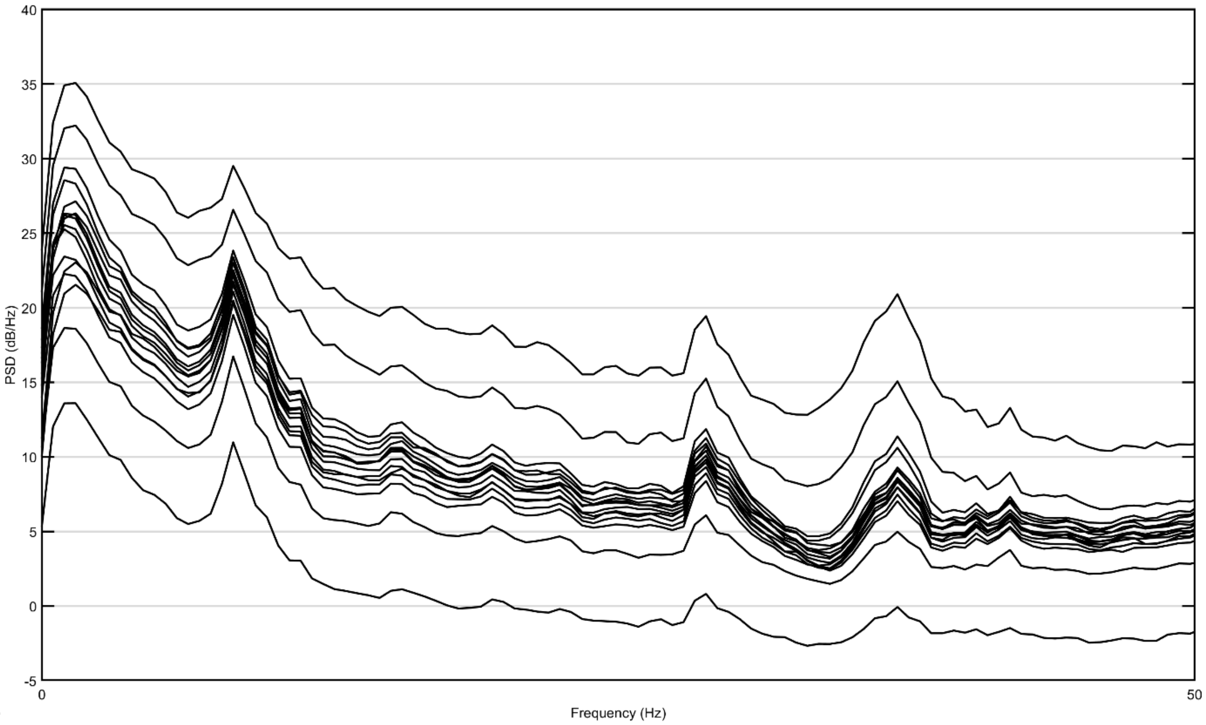

40
35
30
25
20
15
10
5
0
-5
PSD (dB/Hz)
0
50
Frequency (Hz)

(C)

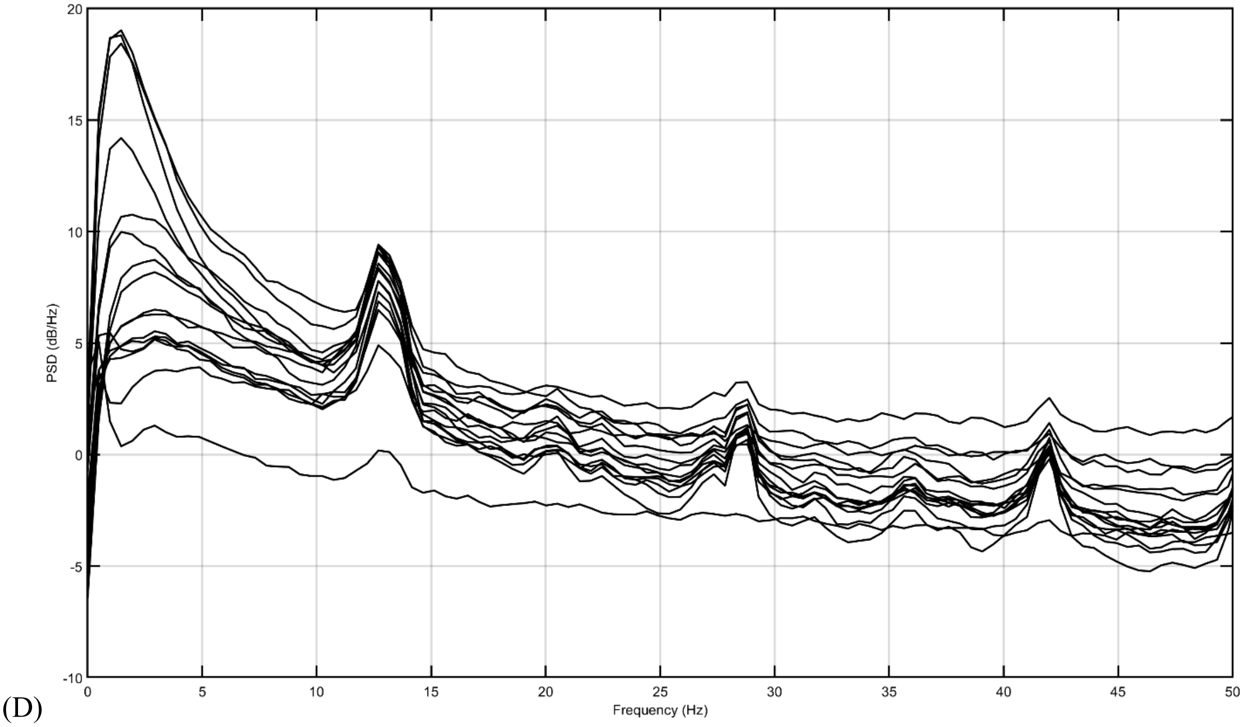

20
15
10
5
0
-5
-10
PSD (dB/Hz)
0
5
10
15
20
25
30
35
40
45
50
Frequency (Hz)

(D)

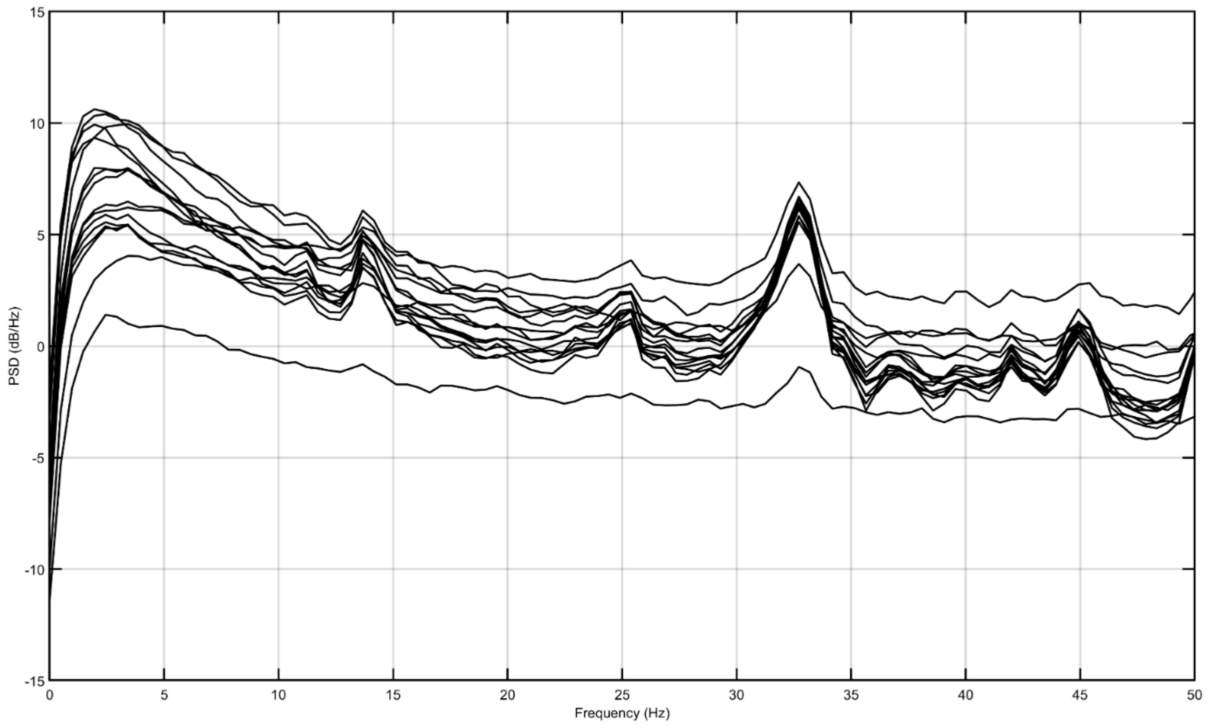
PSD (dB/Hz)
Frequency (Hz)
15
10
5
0
-5
-10
-15
0
5
10
15
20
25
30
35
40
45
50
(E)

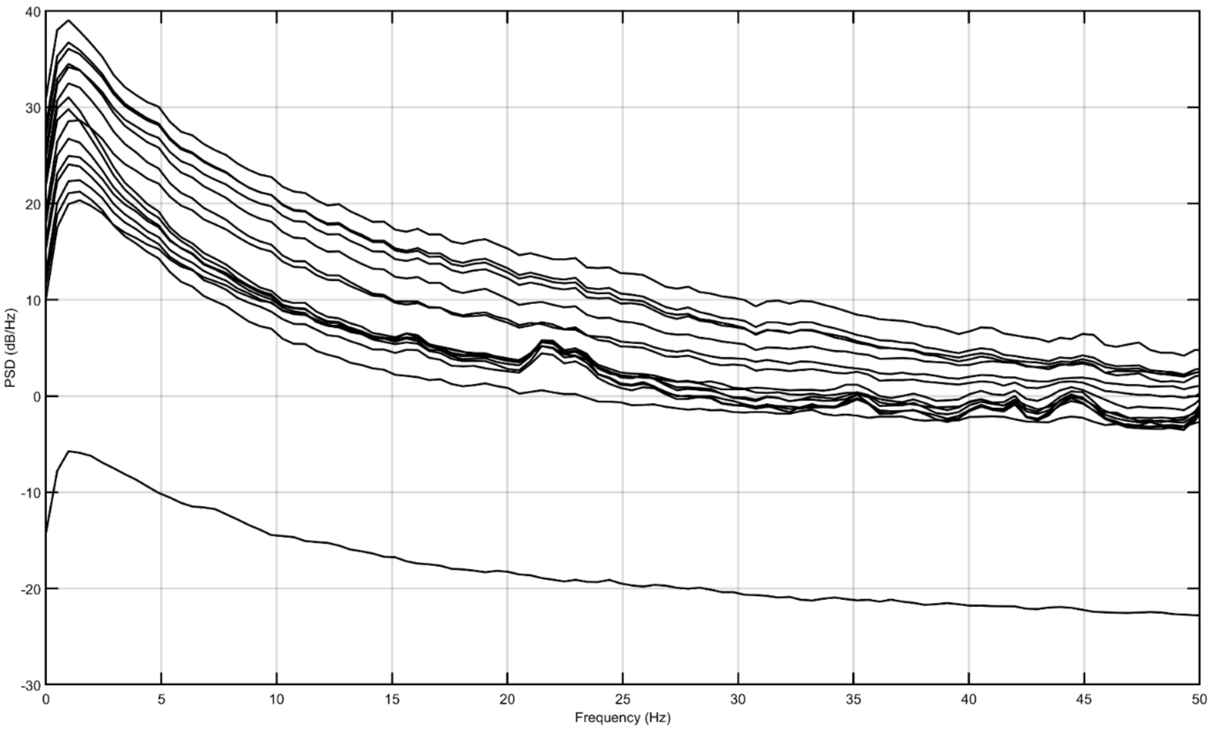
PSD (dB/Hz)
Frequency (Hz)
40
30
20
10
0
-10
-20
-30
0
5
10
15
20
25
30
35
40
45
50
(F)

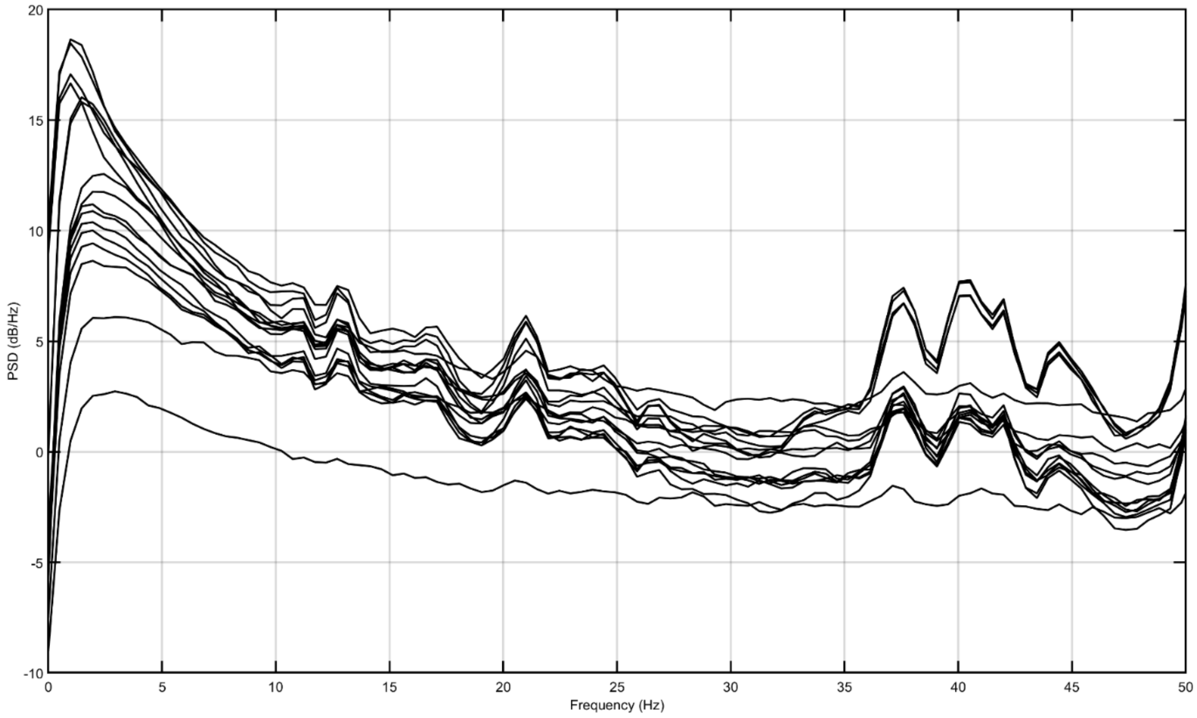


(G)

Figure A2: Power spectral density (PSD) plot for 16 best (most spikes per min) tetrode recordings. (A) PSD for cerebral organoid at 23 days from healthy controls, (B) PSD for cerebral organoid at two months from healthy controls, (C) PSD for cerebral organoid at three months from healthy controls, (D) PSD for cerebral organoid at 34 days from schizophrenia (SCZ) patients, treated with vehicle, (E) PSD for cerebral organoid at 34 days from schizophrenia (SCZ) patients, treated with choline (SCZ+CHO), (F) PSD for cerebral organoid at 34 days from schizophrenia (SCZ) patients, treated with idebenone (SCZ+IDB), (G) PSD for cerebral organoid at 34 days from schizophrenia (SCZ) patients, treated with LCLA (SCZ+LCLA).

## Appendix 3

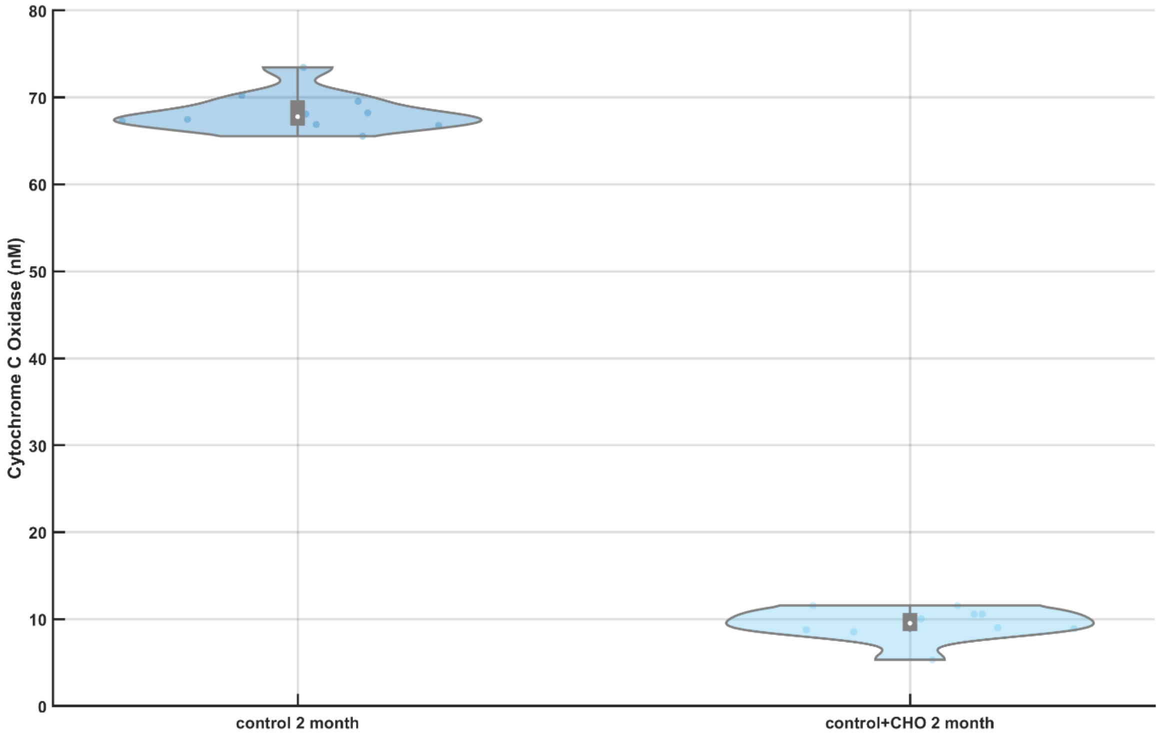


(A)

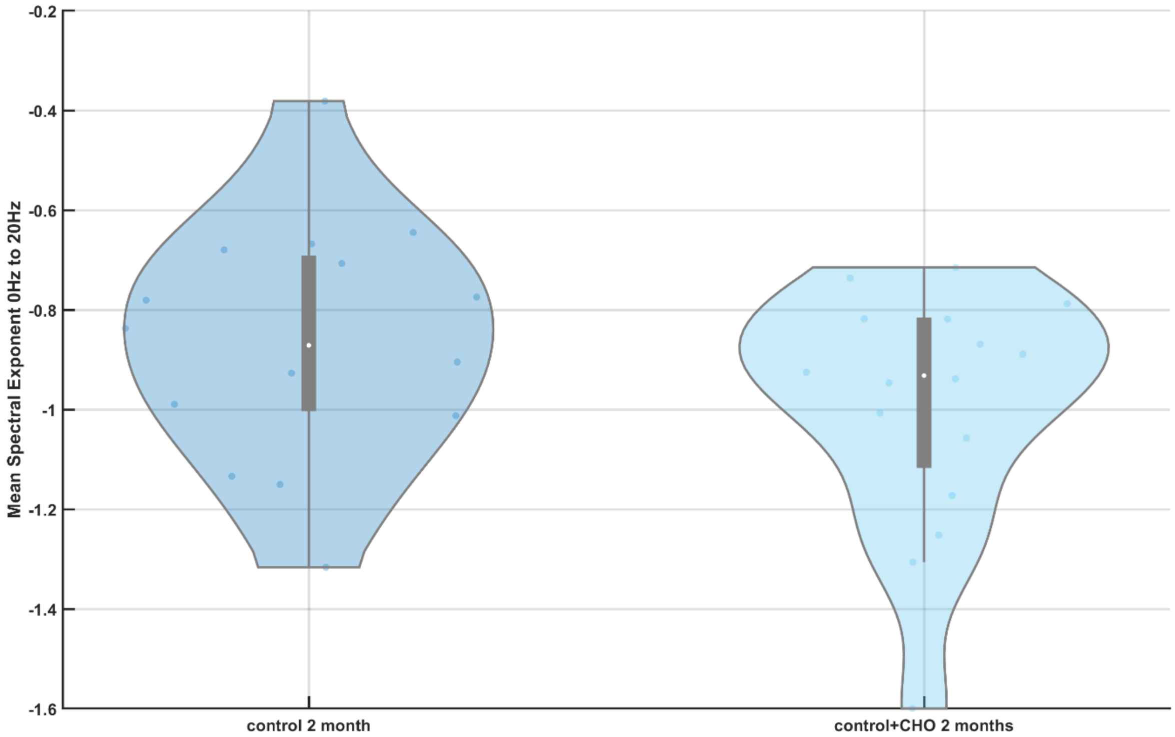


(B)

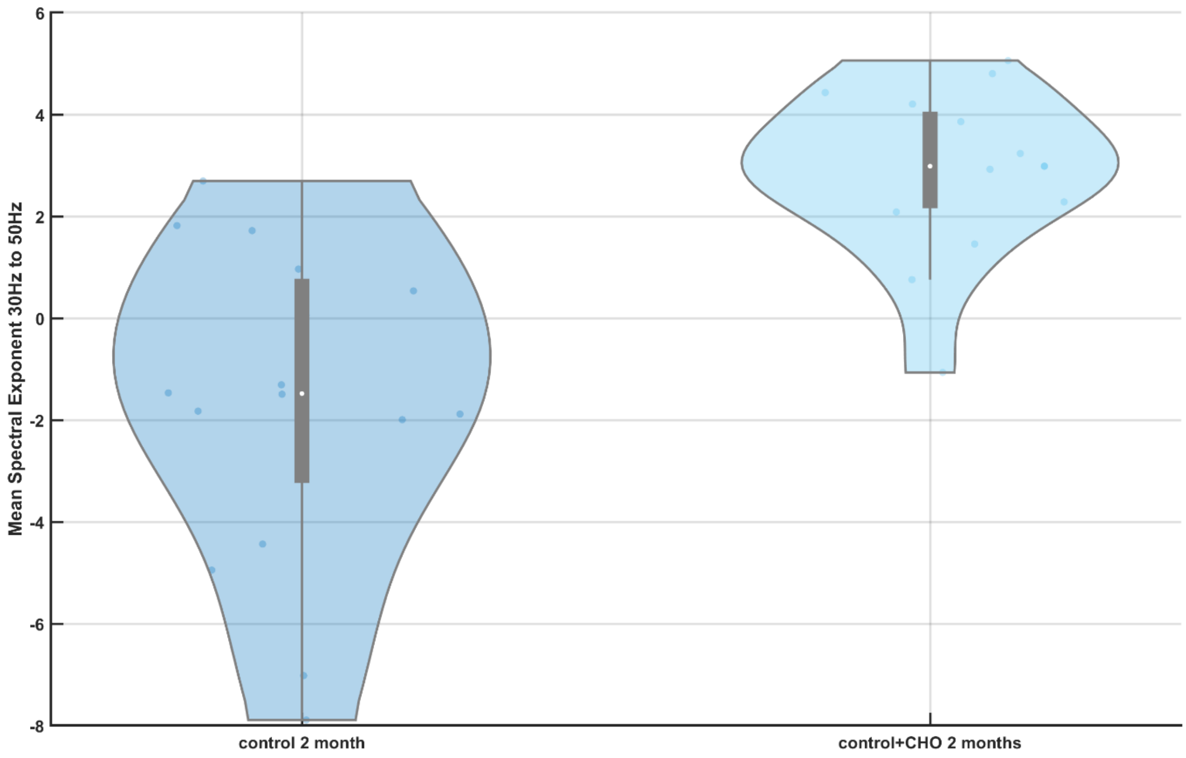


(C)

Figure A3.1: (A) Violin plot of the cytochrome C oxidase (CCO) activity across ten trials in 2 months old cerebral organoids from healthy controls (control) treated with vehicle and the drug, choline (CHO). (B) Violin plot of the spectral exponent in the 1 – 20Hz frequency band computed for 16 best (most spikes per min) tetrode recordings from 2 months old cerebral organoids from healthy controls (control) treated with vehicle and drug, CHO. (C) Violin plot of the spectral exponent in the 30 – 50Hz frequency band computed for 16 best (most spikes per min) tetrode recordings from 2 months old cerebral organoids from healthy controls (control) treated with vehicle and drug, CHO. Violin plot allowed visualization of the distribution of the data and its probability density where the box plot (with median, interquartile range, upper adjacent value, lower adjacent value) is combined with the probability density placed on each side.

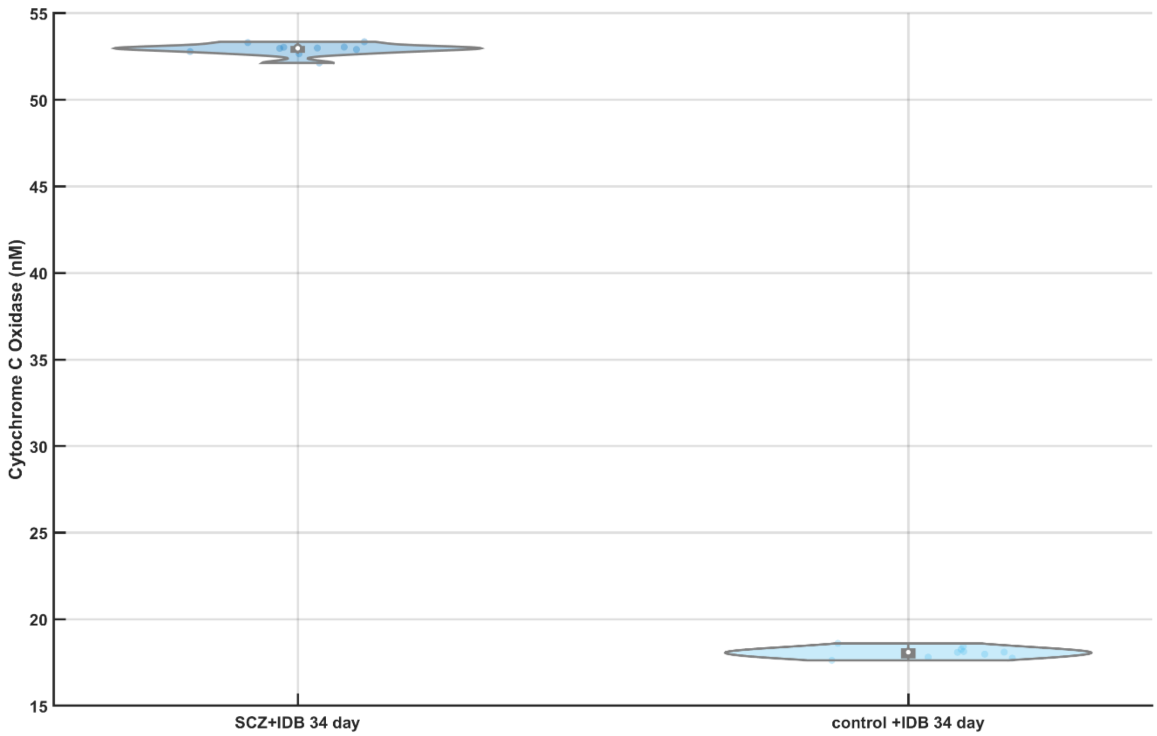

55
50
45
40
35
30
25
20
15
Cytochrome C Oxidase (nM)
SCZ+IDB 34 day
control +IDB 34 day

(A)

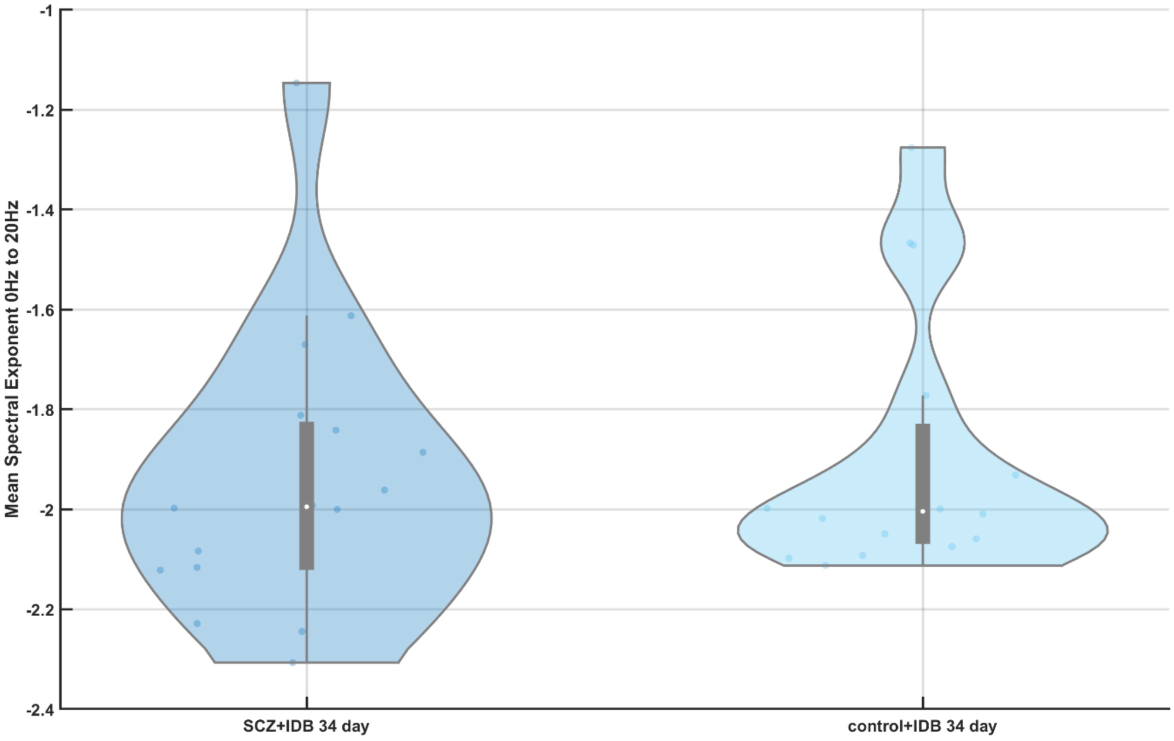

-1
-1.2
-1.4
-1.6
-1.8
-2
-2.2
-2.4
Mean Spectral Exponent 0Hz to 20Hz
SCZ+IDB 34 day
control+IDB 34 day

(B)

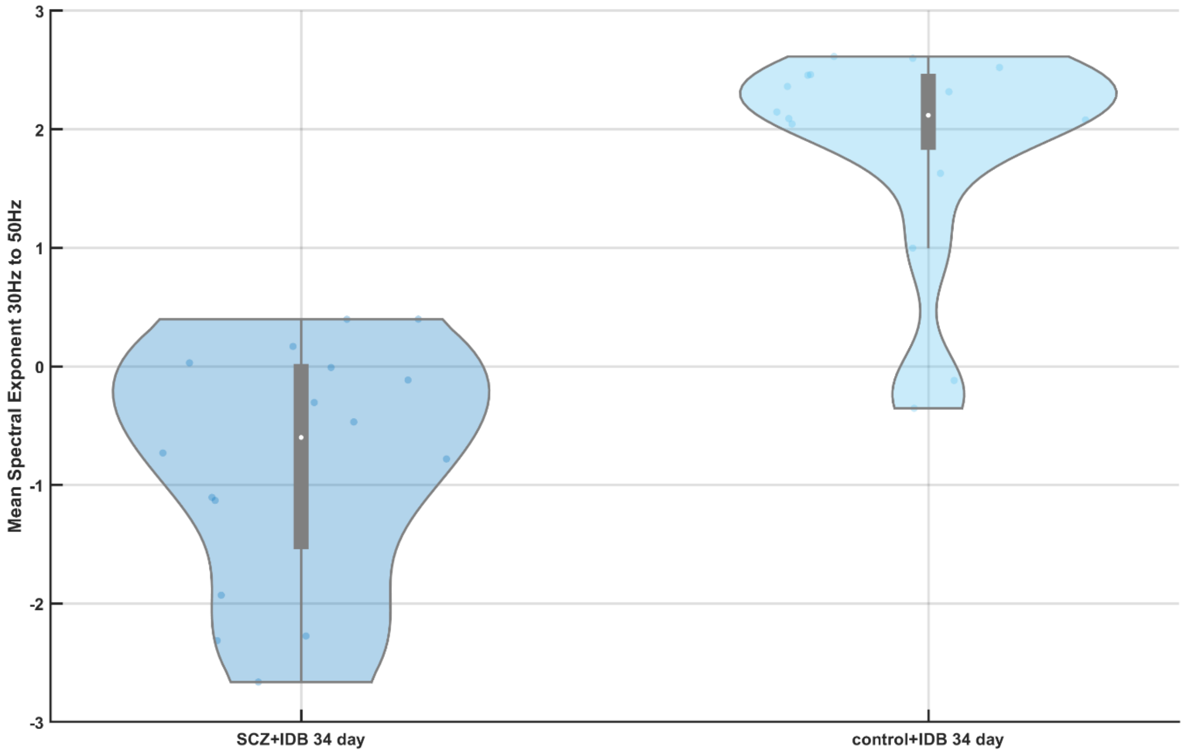


(C)

Figure A3.2: (A) Violin plot of the cytochrome C oxidase (CCO) activity across ten trials in 34 days old cerebral organoids from schizophrenia (SCZ) patients and healthy controls (control) treated with vehicle and drug, idebenone (IDB). (B) Violin plot of the spectral exponent in the 1 – 20Hz frequency band computed for 16 best (most spikes per min) tetrode recordings from 34 days old cerebral organoids from schizophrenia (SCZ) patients and healthy controls (control) treated with vehicle and drug, IDB. (C) Violin plot of the spectral exponent in the 30 – 50Hz frequency band computed for 16 best (most spikes per min) tetrode recordings from 34 days old cerebral organoids from schizophrenia (SCZ) patients and healthy controls (control) treated with vehicle and drug, IDB. Violin plot allowed visualization of the distribution of the data and its probability density where the box plot (with median, interquartile range, upper adjacent value, lower adjacent value) is combined with the probability density placed on each side.

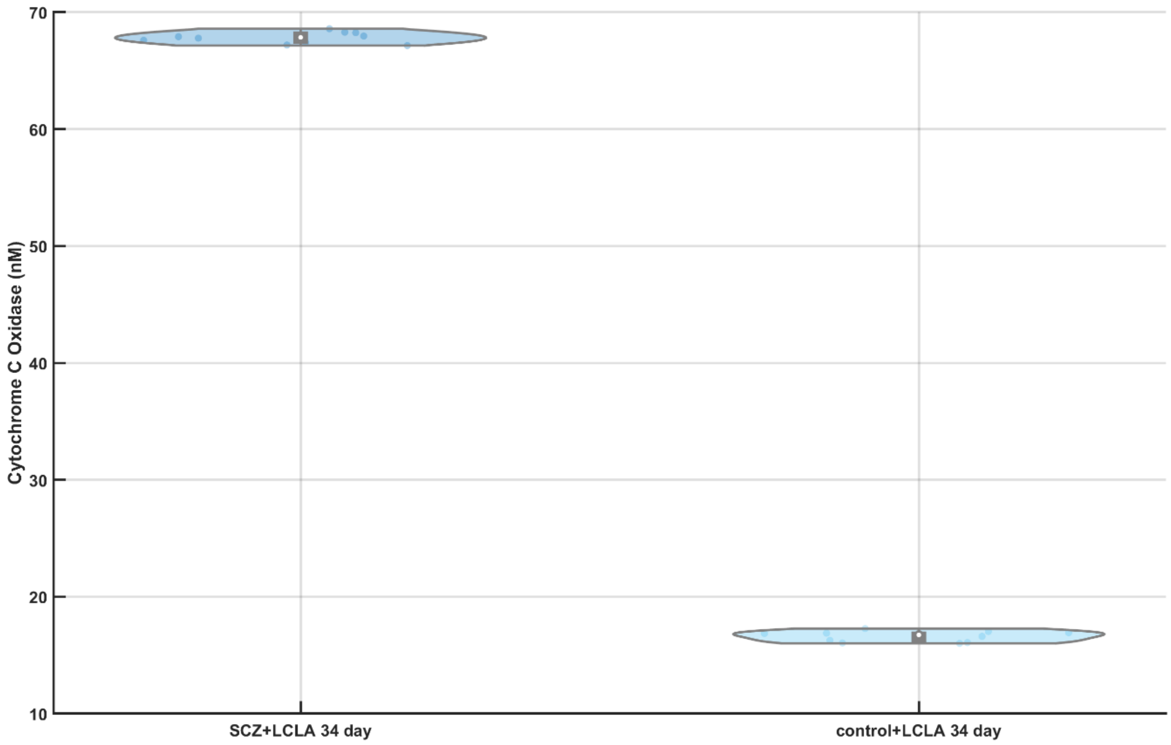

Cytochrome C Oxidase (nM)
70
60
50
40
30
20
10
SCZ+LCLA 34 day
control+LCLA 34 day


(A)

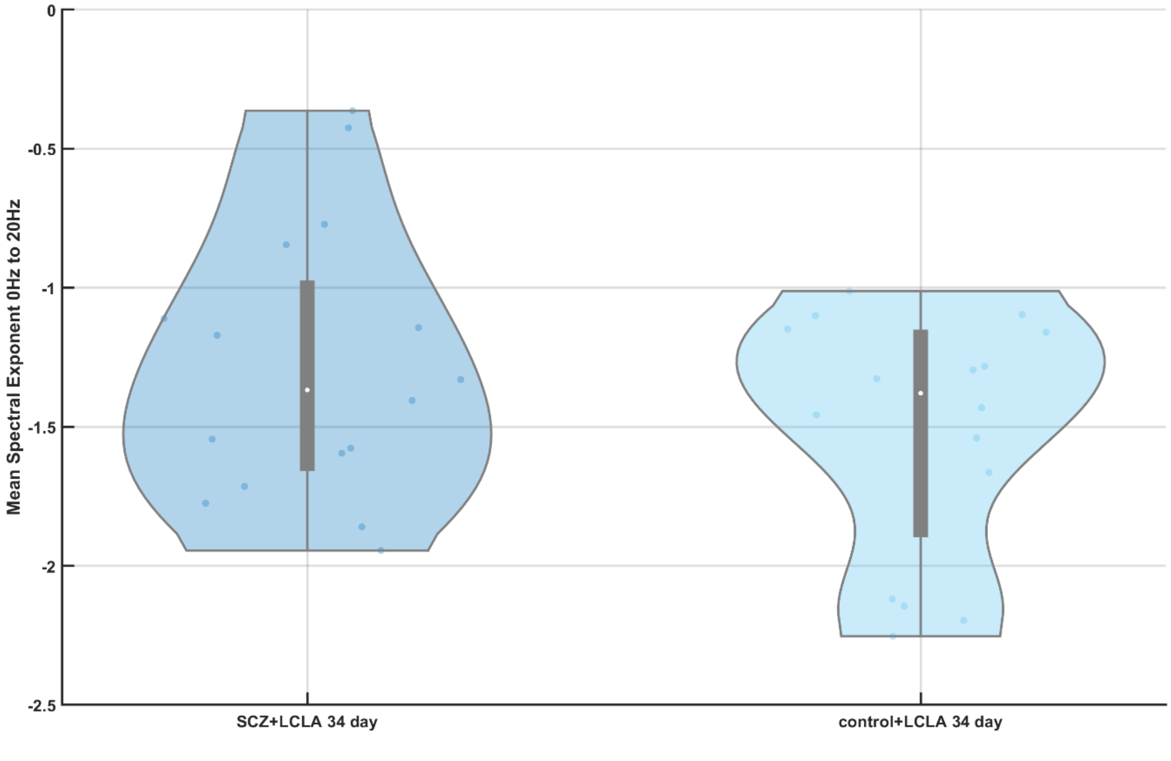

Mean Spectral Exponent 0Hz to 20Hz
0
-0.5
-1
-1.5
-2
-2.5
SCZ+LCLA 34 day
control+LCLA 34 day


(B)

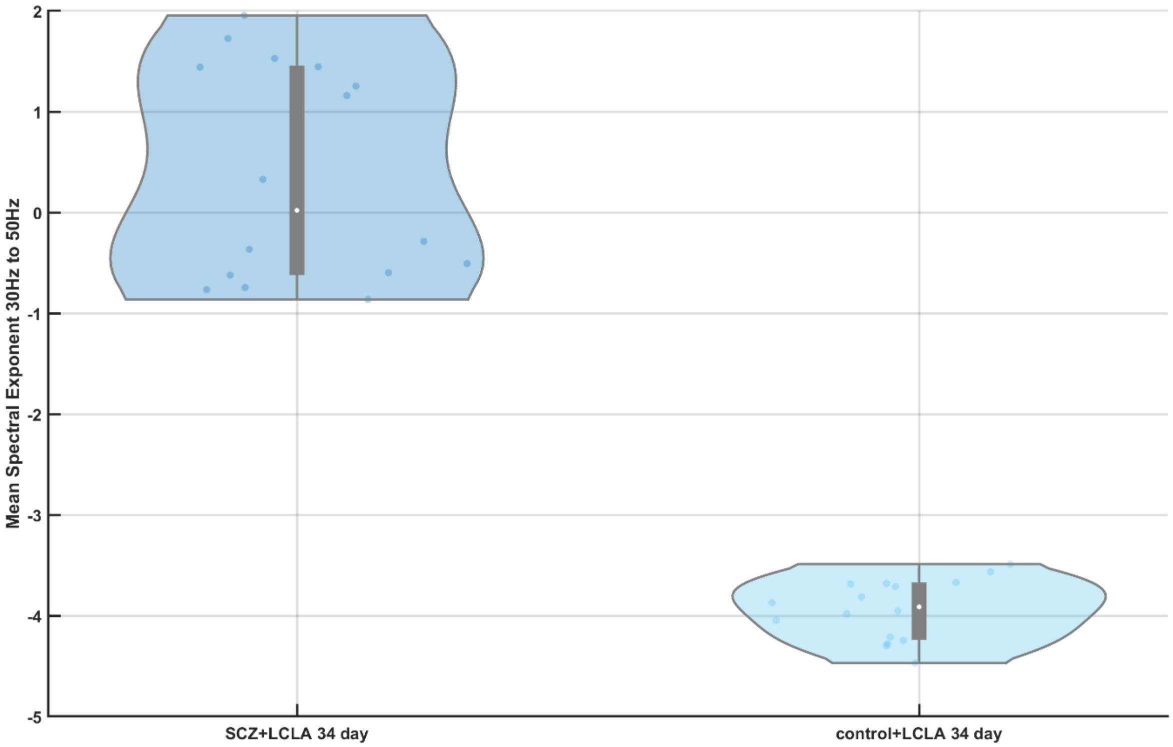


(C)

Figure A3.3: (A) Violin plot of the cytochrome C oxidase (CCO) activity across ten trials in 34 days old cerebral organoids from schizophrenia (SCZ) patients and healthy controls (control) treated with vehicle and drug, R-alpha-lipoic acid plus acetyl-L-carnitine (LCLA). (B) Violin plot of the spectral exponent in the 1 – 20Hz frequency band computed for 16 best (most spikes per min) tetrode recordings from 34 days old cerebral organoids from schizophrenia (SCZ) patients and healthy controls (control) treated with vehicle and drug, LCLA. (C) Violin plot of the spectral exponent in the 30 – 50Hz frequency band computed for 16 best (most spikes per min) tetrode recordings from 34 days old cerebral organoids from schizophrenia (SCZ) patients and healthy controls (control) treated with vehicle and drug, LCLA. Violin plot allowed visualization of the distribution of the data and its probability density where the box plot (with median, interquartile range, upper adjacent value, lower adjacent value) is combined with the probability density placed on each side.

## Appendix 4

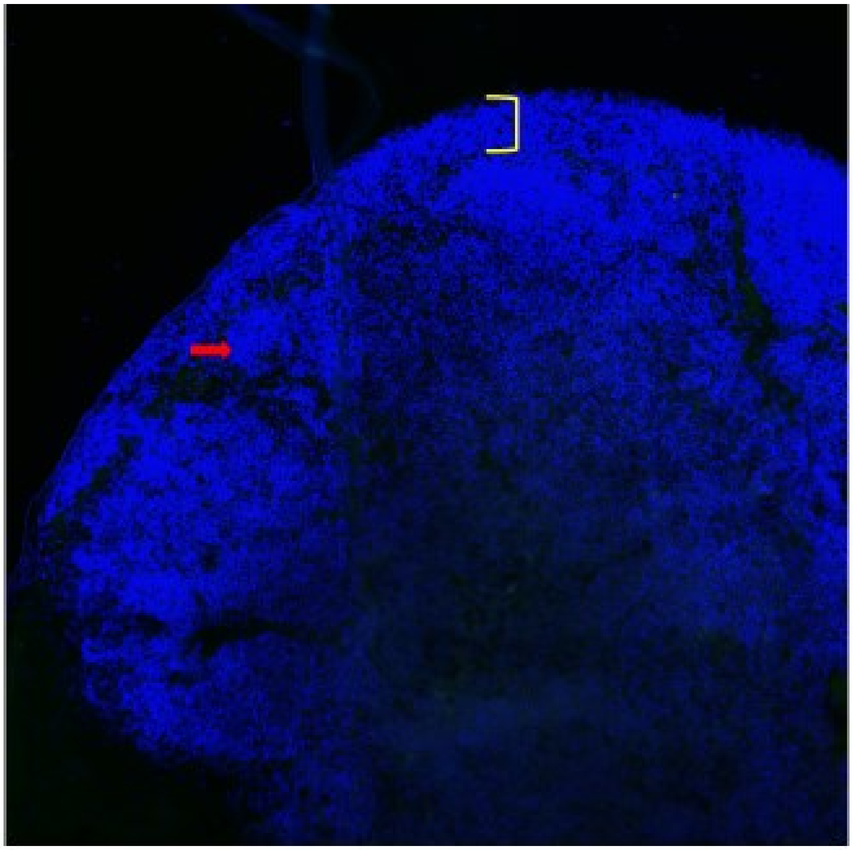

Figure A4: Example of DAPI stained control iPSC cerebral organoid. Red arrow points to one of the rosettes and the yellow arrow points to the cortical zone.
